# Systematic review and meta-analysis of performance of wearable artificial intelligence in detecting and predicting depression

**DOI:** 10.1038/s41746-023-00828-5

**Published:** 2023-05-05

**Authors:** Alaa Abd-Alrazaq, Rawan AlSaad, Farag Shuweihdi, Arfan Ahmed, Sarah Aziz, Javaid Sheikh

**Affiliations:** 1 AI Center for Precision Health, Weill Cornell Medicine-Qatar, Doha, Qatar; 2 College of Computing and Information Technology, University of Doha for Science and Technology, Doha, Qatar; 3 School of Medicine, Leeds Institute of Health Sciences, University of Leeds, Leeds, UK

**Keywords:** Public health, Diagnosis

## Abstract

Given the limitations of traditional approaches, wearable artificial intelligence (AI) is one of the technologies that have been exploited to detect or predict depression. The current review aimed at examining the performance of wearable AI in detecting and predicting depression. The search sources in this systematic review were 8 electronic databases. Study selection, data extraction, and risk of bias assessment were carried out by two reviewers independently. The extracted results were synthesized narratively and statistically. Of the 1314 citations retrieved from the databases, 54 studies were included in this review. The pooled mean of the highest accuracy, sensitivity, specificity, and root mean square error (RMSE) was 0.89, 0.87, 0.93, and 4.55, respectively. The pooled mean of lowest accuracy, sensitivity, specificity, and RMSE was 0.70, 0.61, 0.73, and 3.76, respectively. Subgroup analyses revealed that there is a statistically significant difference in the highest accuracy, lowest accuracy, highest sensitivity, highest specificity, and lowest specificity between algorithms, and there is a statistically significant difference in the lowest sensitivity and lowest specificity between wearable devices. Wearable AI is a promising tool for depression detection and prediction although it is in its infancy and not ready for use in clinical practice. Until further research improve its performance, wearable AI should be used in conjunction with other methods for diagnosing and predicting depression. Further studies are needed to examine the performance of wearable AI based on a combination of wearable device data and neuroimaging data and to distinguish patients with depression from those with other diseases.

## Introduction

Depression is a serious illness that affects ~3.8% of the population worldwide (i.e., 280 million people)^1^. Depression “causes feelings of sadness and/or a loss of interest in activities that were once enjoyed” and can lead to a variety of emotional and physical problems for those affected. Individuals with depression may have a decreased ability to interact and function at home and/or at work^2^. They may experience feelings of sadness, changes in appetite, altered sleep patterns, and/or feelings of fatigue. Depressed individuals may also experience feelings of worthlessness and guilt, poor concentration, and impaired decision-making, as well as being at increased risk of suicide and/or death^2^. If left untreated, it can become disabling and can lead to poor quality of life^2^. One study found that depressed adults had 28 more years of quality-adjusted life expectancy (QALE) than non-depressed adults, resulting in a 28.9-year QALE loss due to depression^3^. Therefore, it is very crucial to detect depression as soon as possible.

Current approaches for the assessment of depression disorders are primarily based on clinical observations of patients’ mental states, clinical history, and self-reported questionnaires (e.g., Patient Health Questionnaire-9 (PHQ-9)) for depression. These methods are subjective, time-consuming, and challenging to repeat. As a result, contemporary psychiatric assessments can be inaccurate and ineffective at assessing depression symptoms in a reliable and personalized manner. Furthermore, shortage of mental health professionals worldwide is one of the largest barriers to detecting depression in its early stages^2,4^. For example, there are 9 psychiatrists per 100,000 people in developed countries^5^. The situation is more concerning in middle to low-income countries, where there are 0.1 psychiatrists for every 1,000,000 in low-income countries^6^. Additionally, traditional methods of capacity building (i.e., increasing the number of trained mental health professionals) may take years to achieve^3^. Another factor that can prevent the early detection of depression is the stigma of being labelled as an individual living with a mental health disorder.

While technology has been implemented in healthcare settings with promising results, there is a need to utilize technologies to overcome the challenges of current approaches in depression assessment. Wearable devices have been one of the technologies used for detecting and predicting depression. Wearable devices are usually sensors worn by individuals to collect and analyze biomarkers or biosignals such as heart rates, physical activities, sleep patterns and quality, blood oxygen, and repository rate. Wearable devices are present in various forms such as watches, bands, jewellery, shoes, and clothing. Wearables can be classified into four categories: on-body devices (fixed directly on the body/skin), near-body devices (fixed close to the body with no direct contact with the body/skin), in-body devices (implantable electronics), and electronic textile (textiles with integrated electronics)^7^. The use of wearables has rapidly increased over the past few years; in 2020, 21% of Americans reported using a smartwatch or fitness tracker, a number which continues to grow^8^. Some countries report as high as 45% of their population using wearables^9^.

Symptoms of depression can be assessed by many parameters collected by wearable devices. Due to the desire for automatic, objective, efficient, and real-time approaches to detect or predict depression, Artificial Intelligence (AI) has been utilized with wearable devices, introducing what we call “Wearable AI”. Wearable AI refers to wearable devices that are paired with AI to analyze a large amount of wearable data and provide personalized feedback. Wearable AI has the potential to provide an early and accurate diagnosis and prediction of depression.

Numerous studies have been published on the performance of wearable devices and AI for detecting depression. Several reviews were conducted to summarize these studies; however, they had the following limitations. Firstly, they focused on wearable devices rather than wearable devices paired with AI^10–13^. Secondly, they only targeted certain age groups such as children and adolescents^11,12^. Thirdly, they did not search relevant databases such as PsychInfo^10,12,13^, IEEE Xplore^10–12^, and ACM Digital Library^10–13^. Fourthly, they focused on a specific type of algorithms (neural networks) and data (e.g., electroencephalogram (EEG) data^13^, self-reported data^14^, and neuroimaging data^15^). Lastly, and most importantly, they were not systematic reviews and did not assess the performance of the wearable AI in detecting depression via either a narrative approach or statistical approach (e.g., meta-analysis)^7,10–13^. Therefore, the need for a systematic review that focuses on the performance of wearable AI in detecting and predicting depression has never been higher. To address the above-mentioned limitations, the current review aimed at examining the performance of wearable AI in detecting and predicting depression.

## Results

### Search results

**Fig. 1:**
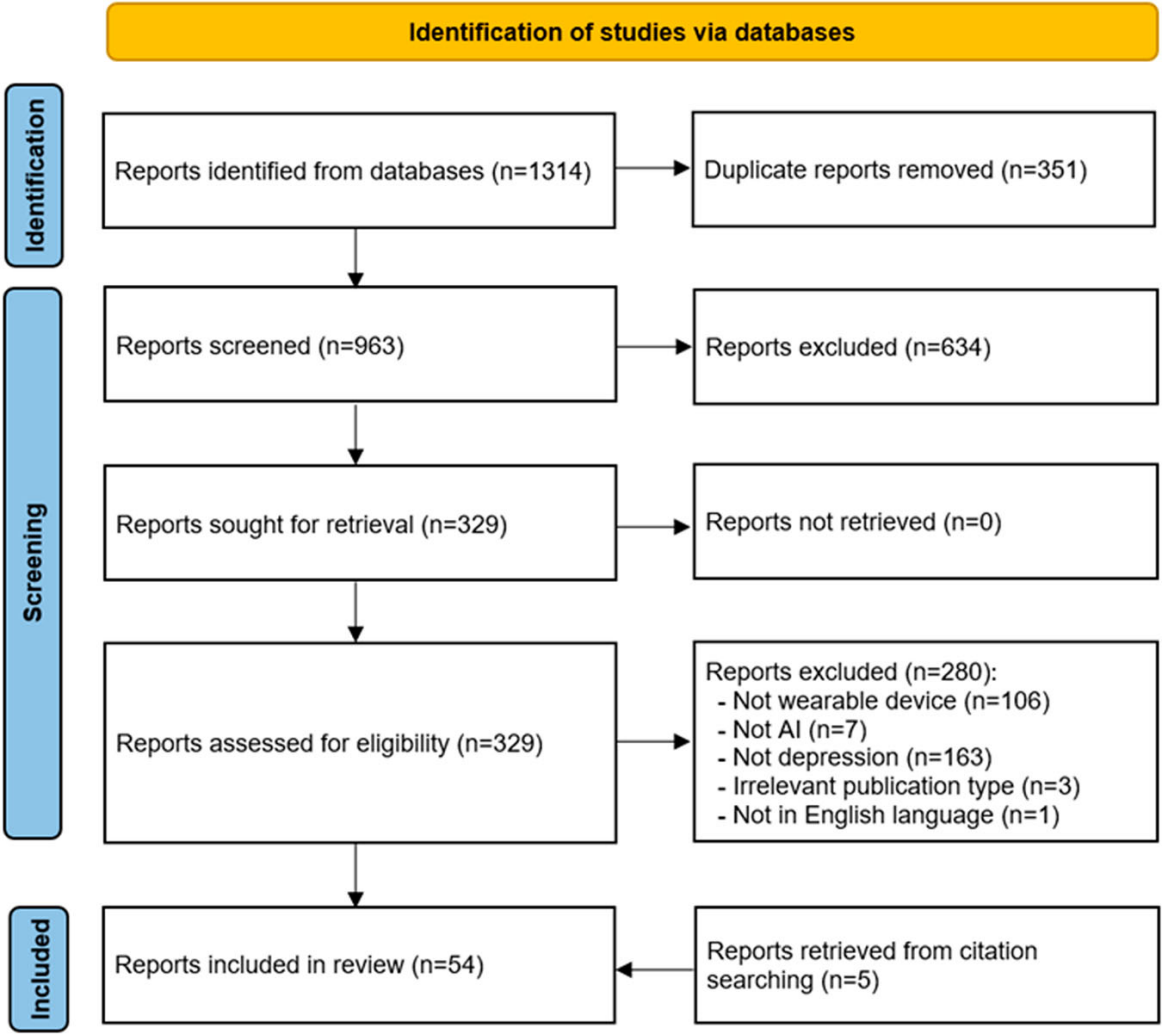
Flow diagram of the study selection process. A total of 1314 publications were retrieved by searching all databases. Of these, 351 duplicates were removed. Screening titles and abstracts of the remaining publications led to excluding 634 citations. By reading the full text of the remaining 329 publications, we excluded 280 publications. Five additional publications were identified by checking the list of the included reviews. In total, 54 publications were included in the current review.

As depicted in Fig. 1, we identified 1314 publications through searching all pre-identified databases. EndNote X9 found and removed 351 duplicates from those publications. Further 634 publications were excluded after screening titles and abstracts of the remaining 963 publications. We retrieved and read the full text of all the remaining 329 publications, and this process led to removing 280 records for several reasons shown in Fig. 1. We identified 5 additional publications relevant to this review by backward and forward reference list checking. Overall, 54 publications were included in the current review^16–69^, and 38 of them were included in the meta-analyses^16–21,24–29,31,35,36,38,41,42,45–50,52,53,56–66,69^.

### Characteristics of included studies

The included studies were published between 2015 and 2022 (Table 1). The year in which the largest number of included studies was published was 2022 (15/54, 27.8%). Studies were carried out in 17 different countries (Table 1), and the country that published the largest number of the included studies was the United States (13/54, 24.1%). The included studies were peer-reviewed journal articles (40/54, 74.1%), conference papers (12/54, 22.2%), and theses (2/54, 3.7%).

**Table 1:** Characteristics of the included studies.

Feature	Number of studies (%)	References
**Year of publication**		
2022	15 (27.8)	^17,22,25,26,33,34,38,41,42,47,50,53,54,57,62^
2021	11 (20.4)	^16,18–20,23,43,46,52,59,63,64^
2020	11 (20.4)	^24,28,36,37,48,49,51,56,60,61,65^
2019	11 (20.4)	^21,27,29,35,39,40,45,55,58,68,69^
2018	4 (7.4)	^30,31,44,67^
2017	1 (1.9)	^32^
2015	1 (1.9)	^66^
**Country of publication**		
USA	13 (24.1)	^20,25,32,34,35,44,50,56,57,64,67,68^
Mexico	7 (13)	^26,29,55,60–62,69^
South Korea	7 (13)	^21–23,38,39,42,51^
Norway	6 (11.1)	^18,27,30,31,36,40^
Japan	4 (7.4)	^28,53,58,65^
United Kingdom	3 (5.6)	^24,33,41^
China	2 (3.7)	^19,37^
India	2 (3.7)	^45,48^
Switzerland	2 (3.7)	^46,47^
Others (Bangladesh, Finland, Italy, Netherlands, Poland, Singapore, Spain, Taiwan)	1 (each) (1.9)	^16,43,49,52,54,59,63,66^
**Type of publication**		
Journal article	40 (74.1)	^16,17,19–26,29,33–37,39,41–45,47–51,54–57,60–66,68,69^
Conference Paper	12 (22.2)	^18,27,28,30–32,38,52,53,58,59,67^
Thesis	2 (3.7)	^40,46^
**Number of participants**		
Mean (Standard Deviation)	315 (826)	^16–69^
Range	8-4036	^16–69^
**Age of participants**		
Mean (Standard Deviation)	39.9 (13)	^16,18,21–27,29–36,38–42,45,47,49,50,52–65,67,69^
Range	15.5-78	^16,18,21–27,29–36,38–42,45,47,49,50,52–65,67,69^
Gender (Female %)		
Mean (Standard Deviation)	60.2 (15.2)	^16,18,21–27,29–42,44,47–50,52–65,67,69^
Range	2.4-100	^16,18,21–27,29–42,44,47–50,52–65,67,69^
**Health conditions**^1^		
Depression	38 (70.4)	^16–21,25–27,29–33,35,36,38–42,44,49–52,54–62,64,65,69^
Healthy	27 (50)	^16,18,20,26,27,29–31,35,36,38,40,41,44,49,51,52,54,55,57–62,65,69^
Any health condition	13 (24.1)	^22–24,28,34,37,46–48,53,63,67,68^
Bipolar	4 (7.4)	^21,42,43,66^
Schizophrenia	1 (1.9)	^62^
Mood swings	1 (1.9)	^45^

Number of participants in the included studies ranged from 8 to 4036, with an average of 315.7 (standard deviation (SD) = 826.1) (Table 1). The mean age of participants was reported in 44 studies and ranged between 15.5 and 78 years, with an average of 39.9 (SD 13). Only 1 of the included studies targeted children (<18 years), and 3 studies focused on only older adults (≥65 years). The percentage of female participants was reported in 46 studies and varied between 2.4% and 100%, with an average of 60.2 (SD 15.2). Half of the studies (27/54, 50%) recruited both patients with depression and healthy individuals. Supplementary Table 1 shows characteristics of each included study.

### Features of wearable AI

The included studies used 30 different wearable devices, but the most common wearable devices used were Actiwatch AW4 (19/54, 35.2%) and Fitbit series (e.g., Fitbit Charge, Fitbit Flex, Fitbit Altra) (14/54, 25.9%) (Table 2). The wearable devices in the included studies were worn on 8 different parts of the body, but the wrist-worn devices were most common in the included studies (50/54, 92.6%).

**Table 2:** Features of wearable AI.

Feature	Number of studies (%)	References
**Wearable device**		
Actiwatch AW4	19 (35.2)	^16,18,26,27,29–31,35,36,40,41,52,55,57,59–62,69^
Fitbit series	14 (25.9)	^20,21,25,28,33,34,42,44,46,47,50,53,63,68^
Empatica series	3 (5.6)	^22,32,56^
Mi Band	2 (3.7)	^19,45^
GENEActiv	2 (3.7)	^43,49^
Others	1 each (1.9)	^17,23,24,38,39,48,49,51,54,58,64–67^
Not reported	1 (1.9)	^37^
**Placement**		
Wrist	50 (92.6)	^16–37,39–47,49–53,55–65,67–69^
Head	1 (1.9)	^48^
Lower back	1 (1.9)	^38^
Fingers	1 (1.9)	^54^
Chest	1 (1.9)	^66^
Waist	1 (1.9)	^23^
Thigh	1 (1.9)	^23^
Ankle	1 (1.9)	^23^
**Aim of AI algorithms**		
Detection	48 (88.9)	^16–20,22–24,26–41,43–46,48–67,69^
Prediction	6 (11.1)	^21,25,34,42,47,68^
Problem-solving approaches		
Classification	44 (81.5)	^16–26,28–31,33,34,36–43,45–49,51–55,57–62,66,68,69^
Regression	5 (9.3)	^32,50,56,64,67^
Classification and regression	5 (9.3)	^27,35,44,63,65^
**AI Algorithms**		
Random Forest	32 (59.3)	^16–19,21,22,25,28–34,36,39,42,43,45,46,48,50,51,54,55,57,59–62,64,69^
Logistic Regression	13 (24.1)	^16,17,19,20,23,25,39,43,45,46,49,54,67^
Support Vector Machine	11 (20.4)	^16,18,19,22,25,30,43,54,55,58,64^
Extreme Gradient Boosting	10 (18.5)	^17,18,22,23,35,46,50,54,63,65^
Decision Tree	8 (14.8)	^18,19,22,30,39,43,48,55^
AdaBoost	8 (14.8)	^20,25,30,32,50,59,64,68^
Convolutional Neural Network	6 (11.1)	^26,27,36,40,41,52^
Ensemble model	6 (11.1)	^32,45–47,52,56^
K-Nearest Neighbours	6 (11.1)	^17,20,22,30,54,55^
Long Short-Term Memory	5 (9.3)	^24,37,38,40,41^
Gradient Boosting	4 (7.4)	^17,20,22,64^
Multilayer Perceptron	3 (5.6)	^22,23,66^
Artificial Neural Network	3 (5.6)	^25,30,59^
Naive Bayes	3 (5.6)	^30,48,55^
Gradient-Boosted Decision Trees	2 (3.7)	^25,46^
Ridge Regression	2 (3.8)	^32,44^
Gaussian Process	2 (3.7)	^30,32^
Linear regression	2 (3.7)	^32,67^
Deep Neural Network	2 (3.7)	^31,36^
elasticNet	2 (3.7)	^34,64^
Support Vector Classifier	2 (3.7)	^17,23^
least Absolute Shrinkage and Selection Operator	2 (3.7)	^20,44^
Others	1 each (1.9)	^18,25,30,32,39,41,48,53,64^
**Dataset source**		
Closed	34 (63)	^19–22,24,25,28,30,32–34,37–39,42–51,53,54,56,58,63–68^
Open	20 (37)	^16–18,23,26,27,29,31,35,36,40,41,52,55,57,59–62,69^
**Data input to AI algorithm**		
Physical activity data	47 (87)	^16–22,25–27,29–45,49–65,67–69^
Sleep data	26 (48.1)	^19–21,25,28,32–34,39,42–47,49–51,53,54,56,63–65,67,68^
Heart rate data	17 (31.5)	^17,19,21,22,24,25,42,44,45,50,51,53,54,56,64,65,67^
Mental health measures	12 (22.2)	^21,25,32,34,39,44,46,47,49,51,54,64^
Smartphone usage data	9 (16.7)	^19,20,32,50,51,54,56,67,68^
Location data	9 (16.7)	^19,20,32,44,50,54,56,67,68^
Social interaction data	8 (14.8)	^19,20,32,50,51,56,67,68^
Light exposure	5 (9.3)	^21,39,42,51,65^
Demographic data	5 (9.3)	^25,46,47,49,59^
Electrodermal activity data	4 (7.4)	^17,22,32,56^
Circadian rhythms	3 (5.6)	^23,49,63^
Skin temperature data	2 (3.7)	^22,65^
Weather data	2 (3.7)	^53,56^
Others	1 each (1.9)	^32,37,43,46–48,51,53,64,66^
**Ground truth assessment**		
MADRS	19 (35.2)	^16,18,26,27,29–31,35,36,40,41,52,55,57,59–62,69^
PHQ-4, -8, and -9	14 (25.9)	^19,22,23,25,33,34,46–48,50,51,63,64,67^
DSM-IV and -5	5 (9.3)	^21,38,44,51,66^
HDRS	5 (9.3)	^32,39,45,56,65^
BDI-II	5 (9.3)	^17,20,37,51,68^
Clinical assessment	2 (3.7)	^42,43^
STAI	2 (3.7)	^24,37^
DASS	2 (3.7)	^24,54^
DAMS	2 (3.7)	^28,53^
QIDS	2 (3.7)	^44,66^
GDS	2 (3.7)	^22,39^
Others	1 each (1.9)	^49,62,66^
Not reported	1 (1.9)	^58^
**Validation approach**		
K-fold cross-validation	25 (46.3)	^17,19,22,25–27,29,30,32,33,38,40,45–47,51–53,56,59,62,63,65–67^
Hold-out cross-validation	22 (40.7)	^21,23,24,26,27,29,32,37,39,41–43,45,51,52,56,58,60,61,66,68,69^
Leave-one-out cross-validation	12 (22.2)	^18,20,27,28,31,32,35,36,44,48,50,68^
Nested cross-validation	5 (9.3)	^16,34,54,57,64^
External validation	2 (3.7)	^49,68^
Time-series cross-validation	1 (1.9)	^54^
Not reported	1 (1.9)	^55^

*BDI-II* Beck Depression Inventory-II, *DASS* Depression, Anxiety and Stress Scale, *DSM* Diagnostic and Statistical Manual of Mental Health, *HDRS* Hamilton Depression Rating Scale, *MADRS* Montgomery-Asberg Depression Rating Scale, *PHQ-9* Patient Health Questionnaire-9, *QIDS* Quick Inventory of Depressive Symptomatology, *STAI* State-Trait-Anxiety-Inventory.

AI in this review was used to detect the current depression status in 48 studies or predict the occurrence or level of depression in the future based on previous and current biosignals in 6 studies (Table 2). Studies used algorithms to solve classification problems (44/54, 81.5%), regression problems (5/54, 9.3%), and both classification and regression problems (5/54, 9.3%). There were 36 different algorithms used in the included studies, but the most commonly used algorithms were Random Forest (RF) (32/54, 59.3%), Logistic Regression (LogR) (13/54, 24.1%), and Support Vector Machine (SVM) (11/54, 20.4%). The included studies used datasets from either closed sources (i.e., collected by authors of the study or obtained from previous studies) (34/54, 63%) or open sources (i.e., public databases) (20/54, 37%). Depresjon was the most common dataset obtained from open sources and used in the included studies (17/20, 85%).

The included studies used >30 types of data to develop the model (Table 2). The most common data used to develop the models were physical activity data (e.g., step counts, calories, metabolic rate) (47/54, 87%), sleep data (e.g., duration and patterns) (26/54, 48.1%), heart rate data (e.g., heart rate, heart rate variability, interbeat interval) (17/54, 31.5%), mental health measures (e.g., depression level, anxiety level, stress level, mood status) (12/54, 22.2%), smartphone usage data (e.g., display on/off, charging activity, number of apps used) (9/54, 16.7%), location data (e.g., latitude, longitude, % of time at home) (9/54, 16.7%), and social interaction (e.g., call and message logs) (8/54, 14.8%).

The included studies identified the ground truth based on 13 different tools, but the most common tool was Montgomery-Asberg Depression Rating Scale (MADRS) (19/54, 35.2%). The included studies used 6 different validation methods for the models (Table 2). The most commonly used validation methods were K-fold cross-validation (25/54, 46.3%) and hold-out cross-validation (22/54, 40.7%). Supplementary Table 2 shows features of wearable AI in each included study.

### Results of risk of bias appraisal

**Fig. 2:**
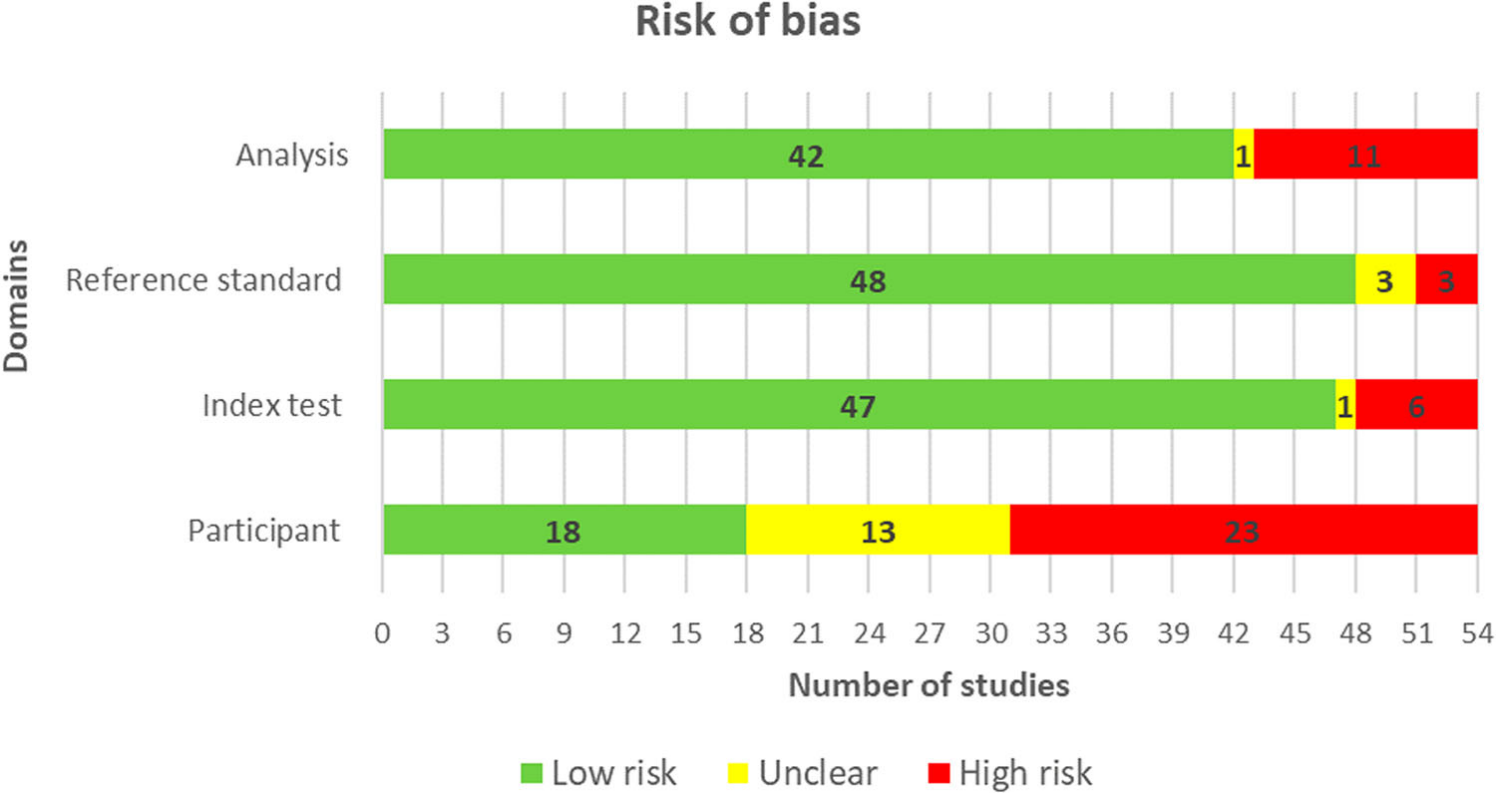
Results of the assessment of risk of bias in the included studies. A modified version of QUADAS-2 was used to assess the risk of bias in the included studies in terms of 4 domains (participants, index test, reference standard, and analysis). Low risk (green) refers to the number of studies that have a low risk of bias in the respective domain. Unclear (yellow) refers to the number of studies that have an unclear risk of bias in the respective domain due to lack of information reported by the study. High risk (Red) refers to the number of studies that have a high risk of bias in the respective domain.

**Fig. 3:**
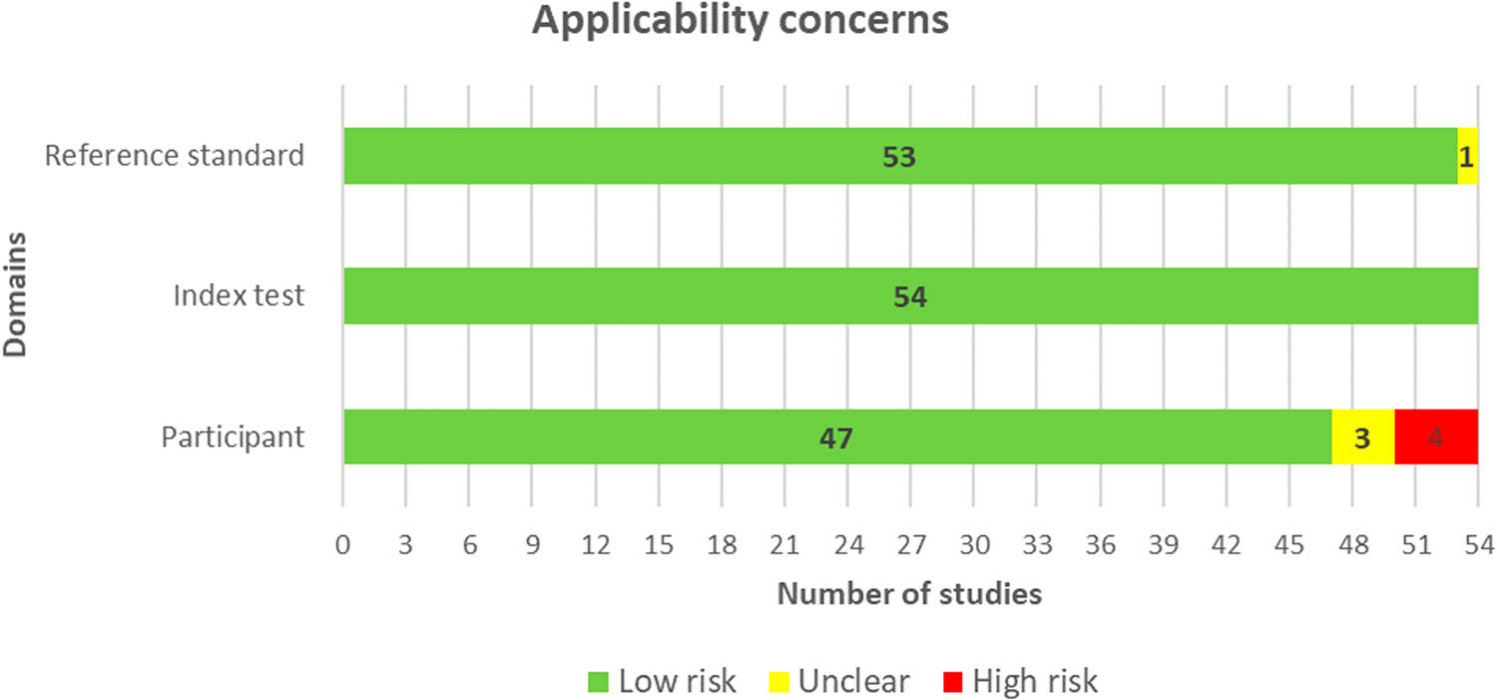
Results of the assessment of applicability concerns in the included studies. A modified version of QUADAS-2 was used to assess the applicability concerns in the included studies in terms of 3 domains (participants, index test, and reference standard). Low risk (green) refers to the number of studies that have a low risk of applicability concerns in the respective domain. Unclear (yellow) refers to the number of studies that have an unclear risk of applicability concerns in the respective domain due to lack of information reported by the study. High risk (Red) refers to the number of studies that have a high risk of applicability concerns in the respective domain.

More than two-thirds of the studies (37/54, 69%) did not provide sufficient information to verify if an appropriate consecutive or random sample of eligible patients was used. Majority of the studies (50/54, 93%) avoided inappropriate exclusions. An adequate balance in the number of patients between the subgroups was used in 73% (34/54) of the studies. Researchers have used an insufficient sample size in 44% (24/54) of the included studies. Thus, the risk of bias owing to the “selection of participants” was rated as low in only 33% (18/54) of the studies (Fig. 2). Concerns regarding the matching between the spectrum of participants and the pre-stated requirements in the review question were rated as low in 87% (47/54) of the studies (Fig. 3).

The AI models were described in detail in 72% (39/54) of the studies. The features (predictors) used in the models were clearly described in almost all studies (53/54, 98%) and were assessed in the same way for all participants in 94% (51/54) of the studies. Features were collected without the knowledge of outcome data in 93% (50/54) of the studies. Therefore, there was a low risk of bias because of the “index test” in 87% (47/54) of the studies (Fig. 2). All the included studies (54/54, 100%) were judged to have low concerns that the definition, assessment, or timing of predictors in the model do not match the review question (Fig. 3).

Researchers in 98% (53/54) of the studies assessed the outcome of interest (i.e., depression level) using appropriate tools. In 94% (51/54) of the studies, the outcome was defined in a similar way for all participants and was determined without knowledge of predictor information. However, only 10 studies (19%) used an appropriate interval between the index test and the reference standard. According to these judgments, the risk of bias because of the “reference standard” was low in 89% (48/54) of the studies (Fig. 2). Nearly all studies (53/54, 98%) were judged to have low concerns that the outcome definition, timing, or determination do not match the review question (Fig. 3).

All participants enroled in the study were included in the data analysis in 65% (35/54) of the studies. In 94% (51/54) of the studies, the data preprocessing was carried out appropriately and the breakdown of the training, validation, and test sets was adequate. The performance of the model was evaluated using suitable measures in 85% (46/54) of the studies. Accordingly, 78% (42/54) of the studies had a low risk of bias in the analysis domain (Fig. 2). Supplementary Table 3 shows reviewers’ judgments about each domain in “risk of bias” and “applicability concerns” for each included study.

### Results of the studies

Meta-analyses were carried out for the highest and lowest results of 4 measures: accuracy, sensitivity, specificity, and RMSE. Further, when applicable, subgroups meta-analyses were performed to assess the performance of wearable AI based on different AI algorithms, aims of AI, used wearable devices, data sources, types of data, and reference standards. The following sections show the above-mentioned results.

#### Accuracy

**Fig. 4:**
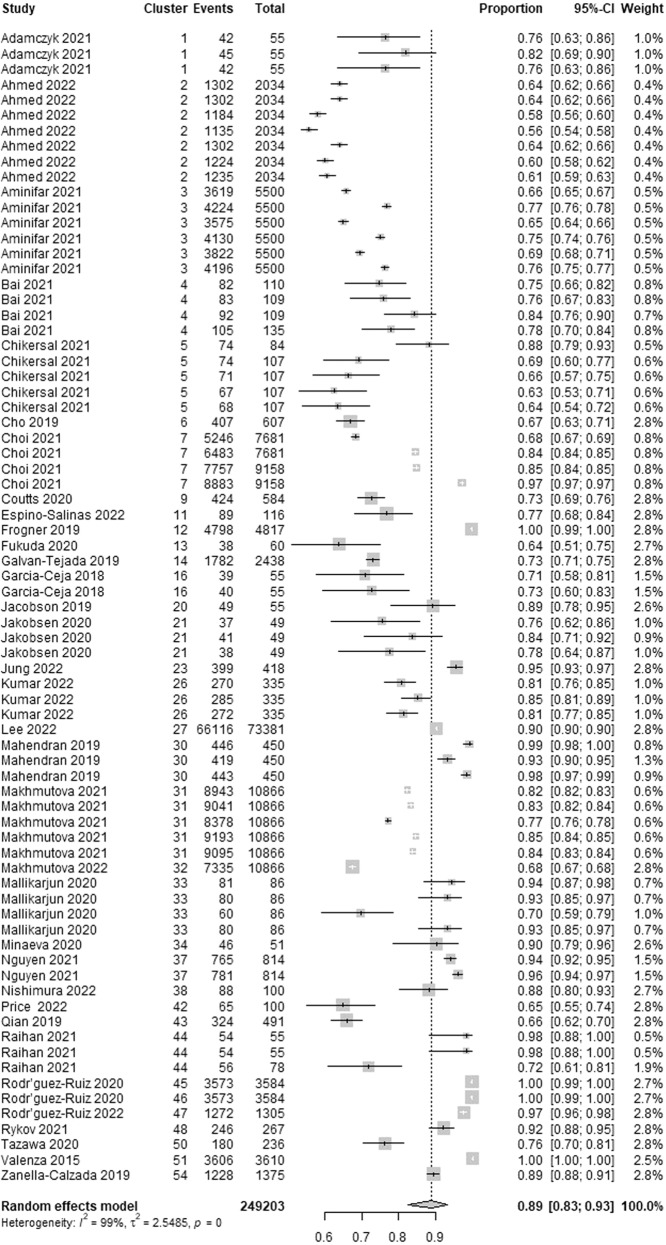
Meta-analysis of the highest accuracy estimates. A total of 75 estimates of the highest accuracy from 35 studies were used in this meta-analysis. The square shape represents the highest accuracy in each study. The rhombus shape represents the pooled estimates of the highest accuracy in all studies. CI Confidence interval. p *p*-value.

Wearable AI accuracy, which is the ability of the AI to correctly classify patients with and without depression, was reported in 35 studies. We identified 75 estimates of accuracy from these studies as many of them reported accuracy for more than one algorithm. The highest accuracy in these studies ranged from 0.56 to 1.00. As presented in Fig. 4, a meta-analysis of the 75 estimates from 249,203 participants in the 35 studies showed a pooled mean accuracy of 0.89 (95% confidence interval (CI) 0.83 to 0.93). The statistical heterogeneity of the evidence was considerable (Cochran’s *p* < 0.001; *I*^2^ = 99.5%). As shown in Supplementary Table 4, subgroup analyses revealed that there is no statistically significant difference in the highest accuracy between subgroups in all groups except for the “algorithms” group (Cochran’s *p* < 0.001).

**Fig. 5:**
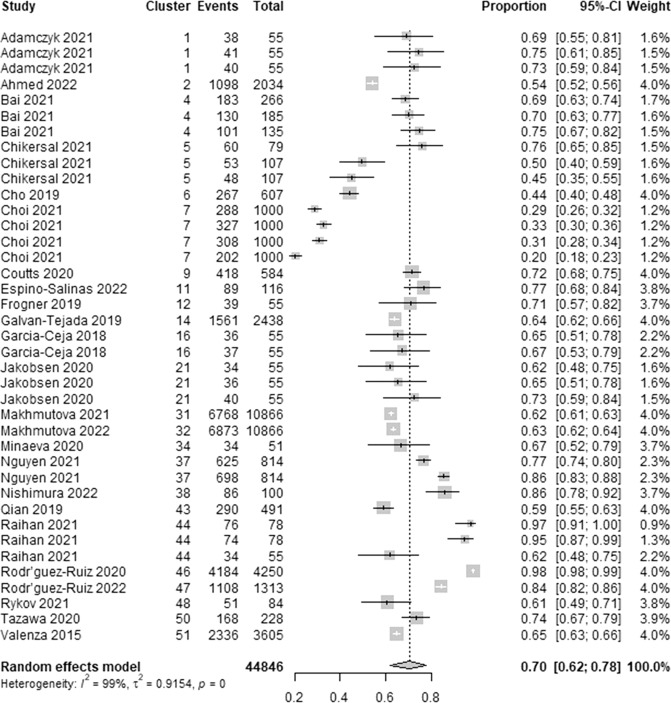
Meta-analysis of the lowest accuracy estimates. A total of 39 estimates of the lowest accuracy from 24 studies were used in this meta-analysis. The square shape represents the lowest accuracy in each study. The rhombus shape represents the pooled estimates of the lowest accuracy in all studies. CI Confidence interval. p *p*-value.

We extracted 39 estimates of the lowest accuracy from 24 studies. The lowest accuracy estimates ranged between 0.20 and 1.00. As demonstrated in Fig. 5, a meta-analysis of the 39 estimates of the lowest accuracy from 44,846 participants in the 24 studies showed a pooled mean of 0.70 (95% CI 0.62 to 0.78). The statistical heterogeneity of the evidence was considerable (Cochran’s *p* < 0.001; *I*^2^ = 98.9%). As shown in Supplementary Table 5, subgroup analyses revealed that there is no statistically significant difference in the lowest accuracy between subgroups in all groups except for the “algorithms” group (Cochran’s *p* < 0.001).

#### Sensitivity

**Fig. 6:**
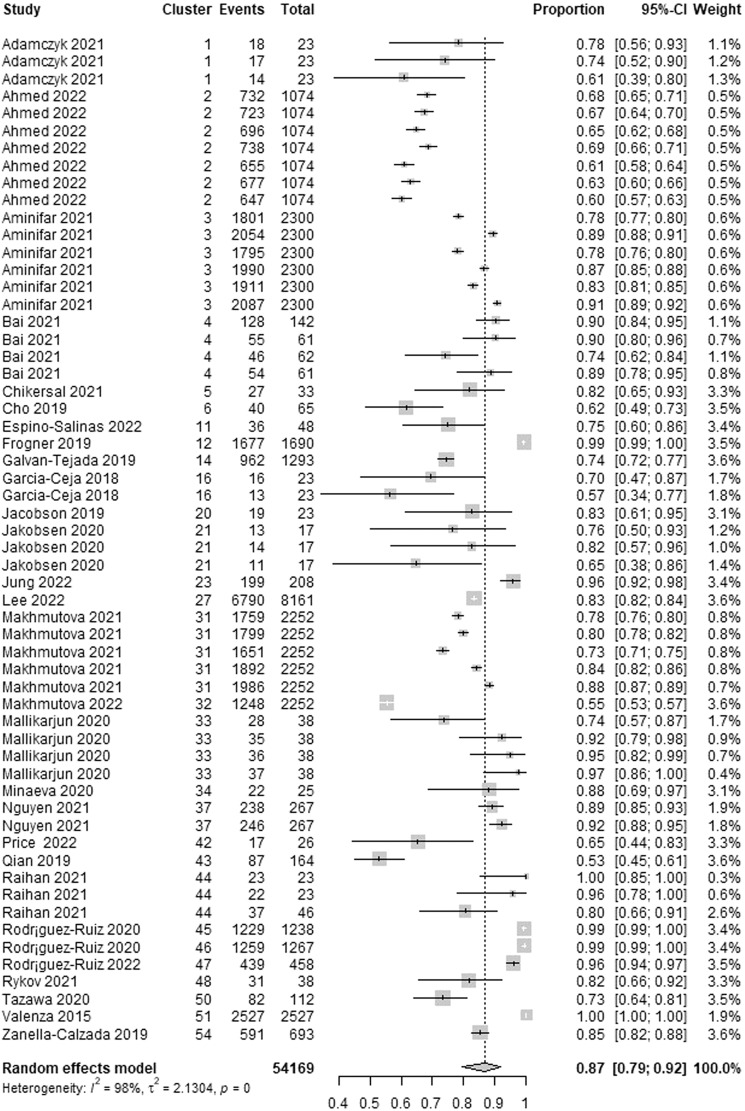
Meta-analysis of the highest sensitivity estimates. A total of 58 estimates of the highest sensitivity from 29 studies were used in this meta-analysis. The square shape represents the highest sensitivity in each study. The rhombus shape represents the pooled estimates of the highest sensitivity in all studies. CI Confidence interval. p *p*-value.

The wearable AI sensitivity, which is the ability of the AI to correctly detect patients with depression, was reported in 29 studies. We identified 58 estimates of sensitivity from these studies because many of them reported sensitivity for more than one algorithm. The highest sensitivity in these studies ranged from 0.53 to 1.00. As presented in Fig. 6, a meta-analysis of the 58 estimates from 54,169 participants in the 29 studies showed a pooled mean sensitivity of 0.87 (95% CI 0.79 to 0.92). The statistical heterogeneity of the evidence was considerable (Cochran’s *p* < 0.001; *I*^2^ = 98.1%). As exhibited in Supplementary Table 6, subgroup analyses revealed that there is no statistically significant difference in the highest sensitivity between subgroups in all groups except for the “algorithms” group (Cochran’s *p* = 0.002).

**Fig. 7:**
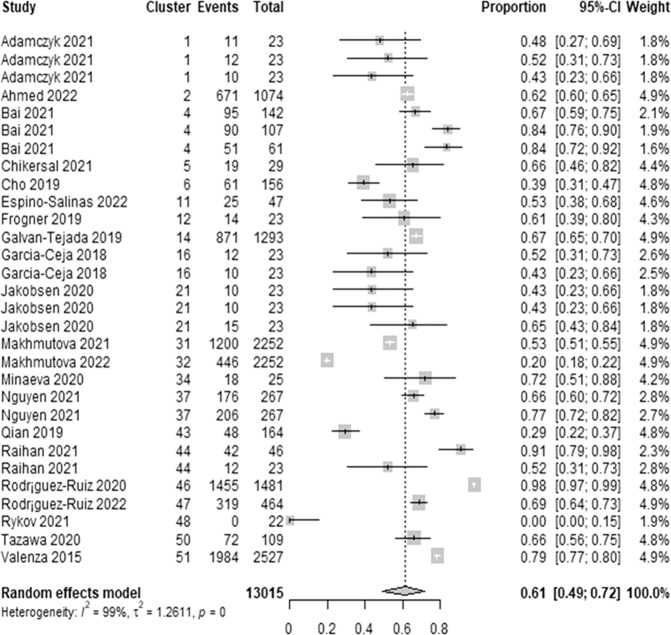
Meta-analysis of the lowest sensitivity estimates. A total of 30 estimates of the lowest sensitivity from 21 studies were used in this meta-analysis. The square shape represents the lowest sensitivity in each study. The rhombus shape represents the pooled estimates of the lowest sensitivity in all studies. CI Confidence interval. p *p*-value.

We extracted 30 estimates of the lowest sensitivity from 21 studies. The lowest sensitivity estimates ranged between 0.00 and 0.98. As demonstrated in Fig. 7, a meta-analysis of the 30 estimates of the lowest sensitivity from 13,015 participants in the 21 studies showed a pooled mean of 0.61 (95% CI 0.49 to 0.72). The statistical heterogeneity of the evidence was considerable (Cochran’s *p* < 0.001; *I*^2^ = 98.6%). As shown in Supplementary Table 7, subgroup analyses revealed that there is no statistically significant difference in the lowest sensitivity between subgroups in all groups except for the “wearable devices” group (Cochran’s *p* = 0.038).

#### Specificity

**Fig. 8:**
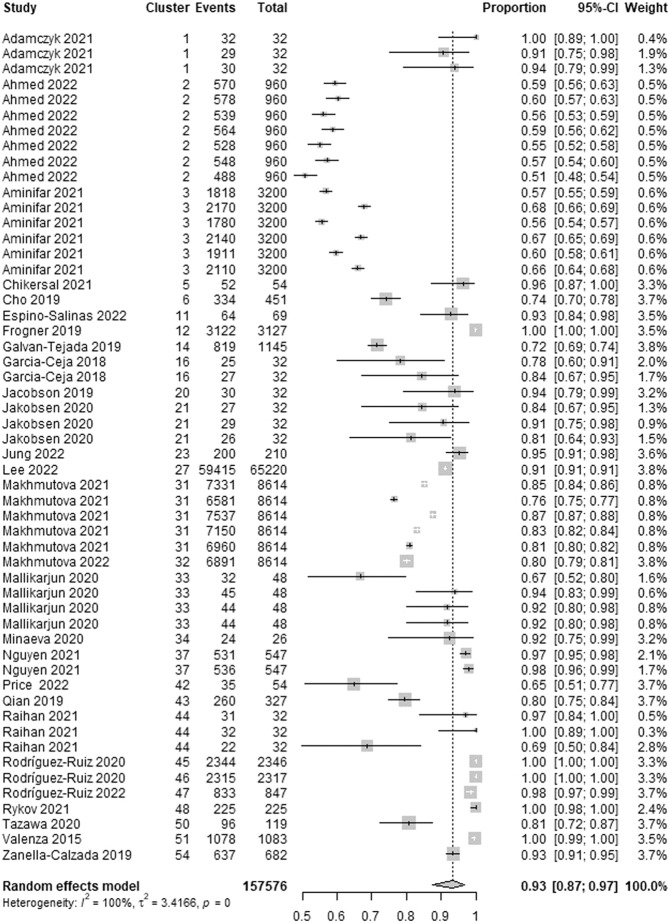
Meta-analysis of the highest specificity estimates. A total of 54 estimates of the highest specificity from 28 studies were used in this meta-analysis. The square shape represents the highest specificity in each study. The rhombus shape represents the pooled estimates of the highest specificity in all studies. CI Confidence interval. p *p*-value.

The wearable AI specificity, which is the ability of the AI to correctly detect patients without depression, was reported in 28 studies. We identified 54 estimates of specificity from these studies given that many of them reported specificity for more than one algorithm. The highest specificity in these studies ranged from 0.51 to 1.00. As presented in Fig. 8, a meta-analysis of the 54 estimates from 157,576 participants in the 28 studies showed a pooled mean specificity of 0.93 (95% CI 0.87 to 0.97). The statistical heterogeneity of the evidence was considerable (Cochran’s *p* < 0.001; *I*^2^ = 99.6%). As shown in Supplementary Table 8, subgroup analyses revealed that there is no statistically significant difference in the highest specificity between subgroups in all groups except for the “algorithms” group (Cochran’s *p* = 0.042).

**Fig. 9:**
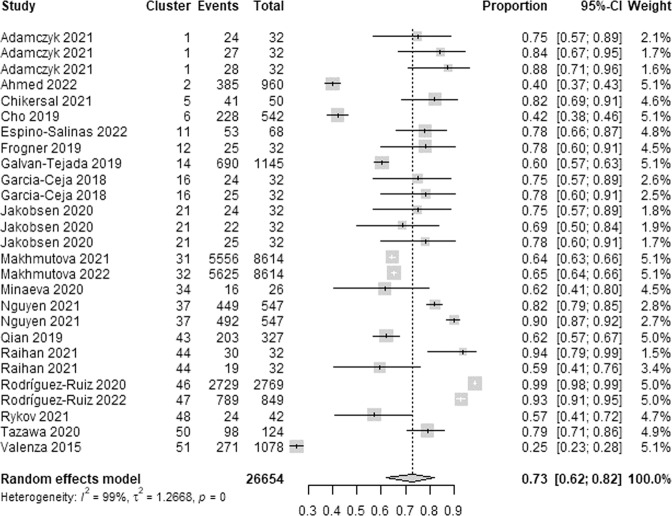
Meta-analysis of the lowest specificity estimates. A total of 27 estimates of the lowest specificity from 20 studies were used in this meta-analysis. The square shape represents the lowest specificity in each study. The rhombus shape represents the pooled estimates of the lowest specificity in all studies. CI Confidence interval. p *p*-value.

We extracted 27 estimates of the lowest specificity from 20 studies. The lowest specificity estimates ranged between 0.25 and 0.99. As demonstrated in Fig. 9, a meta-analysis of the 27 estimates of the lowest specificity from 26,654 participants in the 20 studies showed a pooled mean of 0.73 (95% CI 0.62 to 0.82). The statistical heterogeneity of the evidence was considerable (Cochran’s *p* < 0.001; *I*^2^ = 98.6%). As shown in Supplementary Table 9, subgroup analyses revealed that there is no statistically significant difference in the lowest specificity between subgroups in all groups except for the “algorithms” group (Cochran’s *p* < 0.001) and the “wearable devices” group (Cochran’s *p* = 0.038).

#### Root Mean Suare Error (RMSE)

**Fig. 10:**
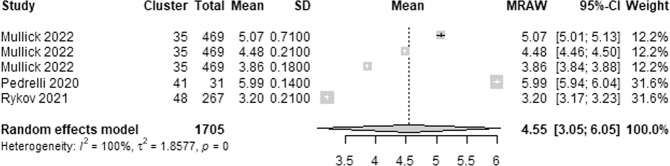
Meta-analysis of the highest RMSE estimates. A total of 5 estimates of the highest RMSE from 3 studies were used in this meta-analysis. The square shape represents the highest RMSE in each study. The rhombus shape represents the pooled estimates of the highest RMSE in all studies. CI Confidence interval. p *p*-value.

The wearable AI RMSE, which estimates the average difference between depression scores predicted by wearable AI and the actual depression scores as assessed by depression assessment tools (e.g., PHQ-9 and HDRS), was reported in 3 studies. We identified 5 estimates of the RMSE from these studies given that one study reported RMSE for 3 algorithms. The highest RMSE in these studies ranged from 3.2 to 6.00. As presented in Fig. 10, a meta-analysis of the 5 estimates from 1,705 participants in the 3 studies showed a pooled mean RMSE of 4.55 (95% CI 3.05 to 6.05). The statistical heterogeneity of the evidence was considerable (Cochran’s *p* < 0.001; *I*^2^ = 100%).

**Fig. 11:**
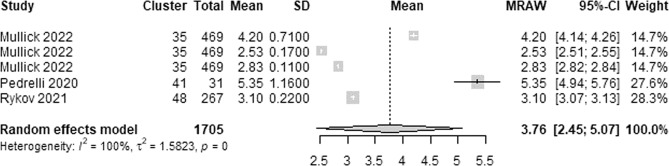
Meta-analysis of the lowest RMSE estimates. A total of 5 estimates of the lowest RMSE from 3 studies were used in this meta-analysis. The square shape represents the lowest RMSE in each study. The rhombus shape represents the pooled estimates of the lowest RMSE in all studies. CI Confidence interval. p *p*-value.

We extracted 5 estimates of the lowest RMSE from 3 studies. The lowest RMSE estimates ranged between 0.11 and 1.16. As shown in Fig. 11, a meta-analysis of the 5 estimates of the lowest RMSE from 1,705 participants in the 3 studies showed a pooled mean RMSE of 3.76 (95% CI 2.45 to 5.07). The statistical heterogeneity of the evidence was considerable (Cochran’s *p* < 0.001; *I*^2^ = 99.9%).

## Discussion

This review examined the performance of wearable AI in detecting and predicting depression. Meta-analyses of estimates from 38 studies revealed AI has a good performance in diagnosing depression using wearable device data, but it is not optimal. Specifically, this review showed that AI could correctly classify patients with and without depression in between 70% from 89% of cases. The review demonstrated that AI has a slightly higher performance in detecting patients without depression (73–93%) than patients with depression (61%-87%). Similarly, this review found that AI has good performance in predicting depression scores using wearable device data, but it is not optimal (RMSE 3.76-4.55).

Subgroup analyses in this review showed that the performance of wearable AI is statistically different between algorithms. To be more precise, AdaBoost outperformed all other algorithms in most analyses. In contrast, logistic regression and decision trees were the worst in most analyses. These results should be interpreted with caution as most of the pooled estimates of the above-mentioned algorithms were based on a few studies (i.e., ≥4) and small sample sizes. Some subgroup analyses found that the performance of wearable AI is affected by the wearable device used to collect data. Specifically, wearable AI has better performance when data is collected by Actiwatch in comparison with Fitbit. This finding should also be interpreted carefully because all studies that used Actiwatch are based on the same dataset (i.e., Depresjon^30^). None of the subgroup analyses showed a statistically significant difference between subgroups in the remaining groups (i.e., aims of AI, data sources, data types, and reference standards).

Similar to the current review, two previous systematic reviews showed that AI has a slightly higher performance in detecting patients without depression (specificity) than patients with depression (sensitivity)^14,15^. However, the two reviews showed pooled sensitivity (80%^14^ and 77%^15^) and specificity (85%^14^ and 78%^15^) that are slightly lower than those in the current review although they were within the range reported in our review. This may be attributed to the fact that the previous reviews focused on the performance of AI based on only self-reported data collected using mobile-based PHQ-9^14^ or neuroimaging data^15^.

From the findings of this review and previous reviews, it seems that AI has a better performance in detecting and predicting depression than in predicting treatment responses in depression. Specifically, a systematic review conducted by Cohen et al. ^70^ found an overall area under the curve of 84%, sensitivity of 77%, and specificity of 79% for AI in predicting response to antidepressant treatment using magnetic resonance imaging (MRI). Another review reported a pooled accuracy of 82% for AI in predicting the outcome of different therapeutic interventions (pharmacological, neuromodulatory, or manual-based psychotherapeutic interventions) using different data types (neuroimaging data, genetic data, and phenomenological data)^71^. A review carried out by Watts et al. ^72^ found a pooled accuracy of 84% for AI in predicting response to pharmacological and nonpharmacological interventions using EEG data. One rationale for AI’s higher performance in detecting and predicting depression rather than predicting treatment responses might be the present research emphasis on diagnostic and predictive analysis of depression rather than prescriptive analysis of depression treatment in this area. More focus should be placed on prescriptive analytic research using wearable devices since these are the gadgets that patients can quickly examine and can cure or reduce the severity of depression on the spot without causing serious effects.

The current review showed that wearable AI is a promising tool for detecting and predicting depression. However, we cannot advocate that wearable AI is ready to be implemented in clinical practices for the following reasons: (1) its performance is not optimal at the present, thus, there is still room for improvement, (2) the sample size was small (≤55) in more than half of the studies (57.4%), (3) about 37% of the studies used publicly available datasets; especially Depresjon, and (4) few studies were judged to have a low risk of bias in all domains. Therefore, wearable AI should be used in conjunction with other methods for diagnosing and predicting depression, such as self-report questionnaires or interviews, to provide a more comprehensive understanding of a patient’s condition.

In this review, AI was not embedded in any of the commercial wearable devices; instead, AI was embedded in a host device (e.g., computers) where the data collected by wearable devices is stored. Thus, we encourage tech companies to develop wearable devices that can detect and predict depression immediately as those that can detect stress (e.g., Fitbit Charge 5, Garmin Instinct Solar 2, Apple Watch Series 7, and Samsung Galaxy Watch 4). We envisage that this could happen in the near future especially as the computing power of wearables increases as new chips are developed and the tech improves. This may encourage researchers to conduct more studies in this area.

None of the included studies used neuroimaging data in addition to wearable device data to detect or predict depression. Several studies showed that AI has a high diagnostic performance (ranging from 92% to 98%) when using neuroimaging data (e.g., diffusion tensor imaging and functional and structural magnetic resonance imaging)^73–77^. Accordingly, future research vistas are to assess the performance of wearable AI in the detection and prediction of depression based on a combination of wearable device data and neuroimaging data.

Most studies (89%) in this review used AI for detecting the current depression status rather than predicting the occurrence or level of depression in the future. Prediction of depression is as important as, or even more important than, detection of depression as this will enable the development of early mental health warning systems and more effective, timely interventions targeted to the individual. Therefore, we urge researchers to conduct further studies on wearable AI for predicting depression.

We noticed that only a few studies in this review used wearable AI to differentiate depression from other disorders (e.g., bipolar, schizophrenia, anxiety, and stress). In clinical practice, complex and error-prone diagnostic processes are usually used to differentiate between various patient groups rather than solely distinguishing them from healthy individuals. Further studies should be conducted to distinguish patients with depression from those with other diseases that have similar signs and symptoms of depression.

As mentioned earlier, the sample size was small (≤55) in more than half of the studies (57.4%). For this reason, potential differences in the performance of wearable AI in subgroup analyses might not have manifested. This might also have prevented researchers to use some algorithms that need a very large sample size to be trained and tested. We urge researchers to conduct further studies with larger samples and over longer periods of time to ensure adequate statistical power as well as to enable the utilization of more complex and efficient algorithms requiring a larger amount of data.

About 61% of the included studies used Fitbit or Actiwatch AW4 to collect biomarkers although there are many other wearable devices in the market. For this reason, most subgroup analyses included only Fitbit or Actiwatch AW4, thereby, differences in the performance of different wearable devices in subgroup analyses might not have manifested. Further, none of the included studies compared the performance of different wearable devices. We recommend researchers use other wearable devices and compare the performance of different wearable devices.

This review cannot comment on (1) the performance of wearable AI in detecting other mental disorders, (2) the performance of wearable AI in predicting outcomes of treatment for depression, and (3) the performance of non-wearable devices, hand-held devices, near-body wearable devices, in-body wearable devices, wearable devices connected with non-wearable devices using wires, and wearable devices that need an expert to apply on users. This is because such disorders, outcomes, and wearable devices were beyond the scope of this review, thereby, our findings may not be generalizable to such contexts. Further, we likely missed some studies given that we restricted our search, for practical constraints, to studies published in the English language from 2015 onwards. Results of our meta-analyses are likely to be overestimated or underestimated given that several included studies were included in the meta-analyses because they did not report results appropriate for the meta-analyses.

Wearable AI is a promising tool for detecting and predicting depression, but it is still in its infancy; meaning, it is not quite ready to be implemented in clinical practice. Until further research improve its performance, wearable AI should be used in conjunction with other methods for diagnosing and predicting depression (e.g., self-report questionnaires or interviews) to provide a more comprehensive understanding of a patient’s condition. Tech companies should embrace the use of AI for the purpose of detecting and predicting depression using wearables. Researchers should examine the performance of wearable AI in the detection and prediction of depression based on a combination of wearable device data and neuroimaging data. Further studies should be conducted to distinguish patients with depression from those with other diseases that have similar signs and symptoms of depression. Wearables utilizing AI for detecting and predicting depression are getting better over time and we will likely see further development in this field via more accurate sensors and improved AI algorithms, we envisage this eventually leading to the possibility of use in clinical practice.

## Methods

### Overview

We adhered to Preferred Reporting Items for Systematic Reviews and Meta-Analyses- Extension for Diagnostic Test Accuracy (PRISMA-DTA)^78^ in reporting this review. Supplementary Table 10 outlines the PRISMA-DTA Checklist for this review. The protocol has been registered in with the International Prospective Register of Systematic Reviews (PROSPERO) (ID: CRD42022367856). The methods used in this review are detailed in the following subsections.

### Search strategy

We identified the relevant studies having searched 8 electronic databases on October 3, 2022: MEDLINE (via Ovid), PsycInfo (via Ovid), EMBASE (via Ovid), CINAHL (via EBSCO), IEEE Xplore, ACM Digital Library, Scopus, and Google Scholar. An automatic search was set up with biweekly alerts for 3 months (ending on January 2, 2023). Only the first 100 hits (i.e.,10 pages) were checked for studies retrieved using Google Scholar in this review, due to the large number of results returned. Reference lists of included studies were checked (i.e., backward reference list checking), and studies that cited the included studies were screened (i.e., forward reference list checking) in order to identify additional studies.

Three experts in digital mental health were consulted whilst developing the search query, furthermore, previous reviews of relevance to the review were checked. Three groups of search terms were used: terms related to AI (e.g., artificial intelligence, machine learning, and deep learning), terms related to wearable devices (e.g., wearable, smartwatch, and smartwatch), and terms related to depression (e.g., depression and mood disorder). The search queries used in this review are highlighted in Supplementary Table 11.

### Study eligibility criteria

This review examined papers that focused on building AI algorithms for depression utilizing wearable device data. We concentrated specifically on all AI algorithms utilized for detecting or predicting depression. We excluded studies that used AI for predicting the outcome of an intervention or treatment for depression. The data acquisition had to be non-invasive on-body wearables such as smartwatches, smart glasses, smart clothes, smart wristbands, and smart tattoos. We excluded studies that used data collected by the following devices: non-wearable devices, hand-held devices (e.g., mobile phones), near-body wearable devices, in-body wearable devices (e.g., implants), wearable devices wired to non-wearable devices, and wearable devices that necessitate expert supervision (e.g., wearable devices composed of many electrodes that need to be placed in very specific points of the body). Studies that used data collected via other methods (e.g., non-wearable devices, questionnaires, and interviews) in addition to wearable devices were considered in this review. To be included in the current review, studies had to assess the performance of the AI algorithms in detecting or predicting depression and report the confusion matrix and/or performance measures (e.g., accuracy, sensitivity, specificity, etc.). We disregarded articles that typically demonstrated a theoretical foundation of AI-powered wearable devices for depression. We accepted journal articles, conference papers, and dissertations published in English since 2015. Reviews, preprints, conference abstracts, posters, protocols, editorials, and comments were not included. There were no constraints on the setting, reference standard, or country of publication.

### Study selection

In the study selection process, we followed three procedures. EndNote X9 was used in the first stage to eliminate duplicates from all retrieved studies. The titles and abstracts of the remaining articles were examined in the second stage. Finally, we read over the whole texts of the studies that were included in the previous stage. The research selection procedure was carried out separately by two reviewers. Disagreements in the second and third phases were settled through dialogue. Cohen’s kappa was used to calculate inter-rater agreement, which was 0.85 for “title and abstract” screening and 0.92 for full-text reading.

### Data extraction

Two reviewers independently extracted data on study meta-data, wearable devices, AI algorithms, and results of studies using Microsoft Excel. Disagreements among the reviewers were overcome through discussion. When the raw data or confusion matrix is reported in the included studies, we calculated all possible performance measures such as accuracy, sensitivity, specificity, and precision. We did not extract results related to the performance of AI algorithms that are based on only non-wearable-device data (e.g., data collected by smartphones or questionnaires). Given that many studies conducted several experiments to test, for example, different numbers of features, data types, validation approaches, and AI techniques, they reported several results for the same performance measure. Therefore, we extracted the lowest and highest results for each performance measure for each algorithm. The data extraction form utilized in this review was trialled with five studies (Supplementary Table 12).

### Risk of bias and applicability appraisal

We modified a well-known risk of bias assessment tool (Quality Assessment of Studies of Diagnostic Accuracy-Revised (QUADAS-2))^79^ by removing some irrelevant criteria and adding other criteria from another relevant tool (the Prediction model Risk Of Bias ASsessment Tool (PROBAST))^80^. Similar to the original QUADAS-2, the modified version evaluates the risk of bias of the included studies in terms of four domains (participants, index test (AI algorithms), reference standard (ground truth), and analysis) whereas it evaluates their applicability to the review question in terms of three domains (participants, index test (AI algorithms), reference standard (ground truth)). Each domain consists of four signalling questions that were tailored to the goal of this review. Based on the answers to these questions, the risk of bias and applicability in the corresponding domain was assessed. Supplementary Table 13 shows the modified version of QUADAS-2, which was pilot tested using four included studies. Two reviewers independently used the modified version to assess the risk of bias and the applicability of the included studies. Disagreements between the two reviewers were settled by discussion.

### Data synthesis

The extracted data were synthesized using narrative and statistical approaches. Knowing that studies or groups of studies reported multiple effect sizes will have a larger influence on the results of the meta-analysis than studies reporting only one effect size. Hence, the risk of biased estimates is high, meaning that the potential dependency between effect sizes, for studies that reported more than one effect size, is needed to be considered in our meta-analysis. Multi-level meta-analysis is a statistical technique used to combine the results of multiple studies while taking into account that data is nested (i.e., the observations are not independent) thereby reducing the likelihood of Type I errors. We, therefore, used a three-level model to analyze the data, where we anticipated a set of studies (level 3), repeated analysis nested within studies (level 2), and a sample of subjects for each analysis (level 1). Using three-level meta-analysis uses three sources of variance: population differences between study population effects, population differences between effects of experiments from the same study, and, finally, sampling variance. We used a random-effects model, assuming a priori significant heterogeneity resulting from diverse study populations and different models. The extracted data was used to compute pooled mean accuracy, sensitivity, specificity, and root mean square error (RMSE). Stratification (subgroup) analysis was conducted based on AI algorithms, aims of AI, wearable devices, data sources, types of data, and reference standards. To assess the degree of heterogeneity and the statistical significance of heterogeneity in the meta-analyzed studies, we computed *I*^2^ and Cochran’s *Q*-test. The presence of heterogeneity in the meta-analyzed studies is indicated by a Cochran’s *p*-value ≤0.05^81^. The degree of heterogeneity was considered insignificant when *I*^2^ ranged from 0% to 40%, moderate when it ranged from 30% to 60%, substantial when it ranged from 50% to 90%, or considerable when it ranged from 75% to 100%^81^. The R version 4.2.2 was used to perform meta-analyses.

### Reporting summary

Further information on research design is available in the Nature Research Reporting Summary linked to this article.

## Supplementary information

Supplementary Information

Reporting Summary

## Data Availability

The data that support the findings of this study are available from the corresponding author upon reasonable request.
