# Supplementary material for: Systematic review and meta-analysis of performance of wearable artificial intelligence in detecting and predicting depression
Source: NPJ Digit Med. 2023 May 5;6:84. doi: 10.1038/s41746-023-00828-5 (PMC10163239; doi:10.1038/s41746-023-00828-5)
Supplement: Supplementary file 1 — Supplementary Information [file 41746_2023_828_MOESM1_ESM.pdf]

Supplementary Table 1: Characteristics of each included study. NR: not reported

Study Ref	Year	Publication type	Country	Sample size	Mean age	Age range	Female %	Participants' health condition
**Adamczyk[1]**	2021	Journal article	Poland	55	40.1	NR	55	Depression, healthy
**Ahmed[2]**	2022	Journal article	United States	142	21.5	18-31	54.9	Depression
**Aminifar[3]**	2021	Conference Paper	Norway	55	40.1	NR	55	Depression, healthy
**Bai[4]**	2021	Journal article	China	261	NR	18-60	NR	Depression
**Chikersal[5]**	2021	Journal article	United States	138	NR	NR	NR	Depression, healthy
**Cho[6]**	2019	Journal article	Korea	55	25.9	21-31	49.1	Depression, bipolar
**Choi[7]**	2021	Journal article	Korea	1552	42.1	NR	55.1	General
**Choi[8]**	2022	Journal article	Korea	14	76	65-86	85.7	General
**Coutts[9]**	2020	Journal article	United Kingdom	668	21.9	18-69	71.2	General
**Dai[10]**	2022	Journal article	United States	89	47.1	NR	76.4	Depression
**Espino-Salinas[11]**	2022	Journal article	Mexico	55	40.1	NR	55	Depression, healthy
**Frogner[12]**	2019	Conference Paper	Norway	55	40.1	NR	55	Depression, healthy
**Fukuda[13]**	2020	Conference Paper	Japan	60	NR	NR	NR	General
**Galvan-Tejada[14]**	2019	Journal article	Mexico	55	40.1	NR	55	Depression, healthy
**Garcia-Ceja[15]**	2018	Conference Paper	Norway	55	40.1	NR	55	Depression, healthy
**Garcia-Ceja[16]**	2018	Conference Paper	Norway	55	40.1	NR	55	Depression, healthy
**Ghandeharioun[17]**	2017	Conference Paper	United States	12	37	20-73	75	Depression
**Griffiths[18]**	2022	Journal article	United Kingdom	17	46.8	21-69	79	Depression
**Horwitz[19]**	2022	Journal article	United States	2459	27.6	NR	55.1	General
**Jacobson[20]**	2019	Journal article	United States	55	40.1	NR	55	Depression, healthy
**Jakobsen[21]**	2020	Journal article	Norway	55	40.1	NR	55	Depression, healthy
**Jin[22]**	2020	Journal article	China	60	NR	18-26	50	General
**Jung [23]**	2022	Conference Paper	Korea	45	76.7	>64	66	Depression, healthy
**Kim[24]**	2019	Journal article	Korea	47	78	NR	94	Depression
**Kulam[25]**	2019	Thesis	Norway	55	40.1	NR	55	Depression, healthy
**Kumar[26]**	2022	Journal article	United Kingdom	55	40.1	NR	55	Depression, healthy
**Lee [27]**	2022	Journal article	Korea	270	23.3	NR	54.4	Depression, bipolar
**Llamocca[28]**	2021	Journal article	Spain	17	NR	NR	NR	Bipolar
**Lu[29]**	2018	Journal article	United States	103	NR	18-25	76.7	Depression, healthy
**Mahendran[30]**	2019	Journal article	India	450	40	NR	NR	Mood swings
**Makhmutova[31]**	2021	Thesis	Switzerland	4036	37.2	18-85	73.7	General
**Makhmutova[32]**	2022	Journal article	Switzerland	4036	37.2	18-85	73.7	General

**Mallikarjun**[33]	2020	Journal article	India	86	NR	NR	100	General
**Minaeva**[34]	2020	Journal article	Netherlands	179	46.5	NR	64	Depression, healthy
**Mullick**[35]	2022	Journal article	United States	55	15.5	12-18	74.5	Depression
**Narziev**[36]	2020	Journal article	Korea	20	NR	NR	NR	Depression, healthy
**Nguyen**[37]	2021	Conference Paper	Taiwan	55	40.1	NR	55	Depression, healthy
**Nishimura**[38]	2022	Conference Paper	Japan	100	42.1	NR	37	General
**Opoku Asare**[39]	2022	Journal article	Finland	54	43	24-68	55.6	Depression, healthy
**Pacheco-Gonzalez**[40]	2019	Journal article	Mexico	55	40.1	NR	55	Depression, healthy
**Pedrelli**[41]	2020	Journal article	United States	31	33.7	19-73	74	Depression
**Price** [42]	2022	Journal article	United States	55	40.1	NR	55	Depression, healthy
**Qian**[43]	2019	Conference Paper	Japan	83	38.4	22-58	2.4	Depression, healthy
**Raihan**[44]	2021	Conference Paper	Bangladesh	55	40.1	NR	55	Depression, healthy
**Rodríguez-Ruiz**[45]	2020	Journal article	Mexico	55	40.1	NR	55	Depression, healthy
**Rodríguez-Ruiz**[46]	2020	Journal article	Mexico	55	40.1	NR	55	Depression, healthy
**Rodríguez-Ruiz**[47]	2022	Journal article	Mexico	109	40.8	NR	48.8	Depression, healthy, schizophrenia
**Rykov**[48]	2021	Journal article	Singapore	267	33	21-64	63.7	General
**Shah**[49]	2021	Journal article	United States	14	21.6	NR	71.4	Depression
**Tazawa**[50]	2020	Journal article	Japan	86	60.2	NR	46.5	Depression, healthy
**Valenza**[51]	2015	Journal article	Italy	8	NR	NR	NR	Bipolar
**Wang**[52]	2018	Conference Paper	United States	83	20.1	NR	51.8	General
**Xu**[53]	2019	Journal article	United States	350	NR	NR	NR	General
**Zanella-Calzada**[54]	2019	Journal article	Mexico	55	40.1	NR	55	Depression, healthy

1 Adamczyk, J. & Malawski, F. Comparison of Manual and Automated Feature Engineering for Daily Activity Classification in Mental Disorder Diagnosis. *Computing and Informatics* **40**, 850–879-850–879 (2021).

2 Ahmed, A. *et al.* Investigating the Feasibility of Assessing Depression Severity and Valence-Arousal with Wearable Sensors Using Discrete Wavelet Transforms and Machine Learning. *Information* **13**, 406 (2022).

3 Aminifar, A., Rabbi, F., Pun, V. K. I. & Lamo, Y. Monitoring Motor Activity Data for Detecting Patients' Depression Using Data Augmentation and Privacy-Preserving Distributed Learning. *Annu Int Conf IEEE Eng Med Biol Soc* **2021**, 2163-2169, doi:10.1109/embc46164.2021.9630592 (2021).

4 Bai, R. *et al.* Tracking and Monitoring Mood Stability of Patients With Major Depressive Disorder by Machine Learning Models Using Passive Digital Data: Prospective Naturalistic Multicenter Study. *JMIR Mhealth Uhealth* **9**, e24365, doi:10.2196/24365 (2021).

5 Chikersal, P. *et al.* Detecting Depression and Predicting its Onset Using Longitudinal Symptoms Captured by Passive Sensing: A Machine Learning Approach With Robust Feature Selection. *ACM Trans. Comput.-Hum. Interact.* **28**, Article 3, doi:10.1145/3422821 (2021).

6 Cho, C. H. *et al.* Mood Prediction of Patients With Mood Disorders by Machine Learning Using Passive Digital Phenotypes Based on the Circadian Rhythm: Prospective Observational Cohort Study. *J Med Internet Res* **21**, e11029, doi:10.2196/11029 (2019).

7 Choi, J. G., Ko, I. & Han, S. Depression Level Classification Using Machine Learning Classifiers Based on Actigraphy Data. *IEEE Access* **9**, 116622-116646, doi:10.1109/ACCESS.2021.3105393 (2021).

8 Choi, J., Lee, S., Kim, S., Kim, D. & Kim, H. Depressed Mood Prediction of Elderly People with a Wearable Band. *Sensors (Basel)* **22**, doi:10.3390/s22114174 (2022).

9 Coutts, L. V., Plans, D., Brown, A. W. & Collomosse, J. Deep learning with wearable based heart rate variability for prediction of mental and general health. *J Biomed Inform* **112**, 103610, doi:10.1016/j.jbi.2020.103610 (2020).

10 Dai, R. *et al.* Multi-Task Learning for Randomized Controlled Trials: A Case Study on Predicting Depression with Wearable Data. *Proc. ACM Interact. Mob. Wearable Ubiquitous Technol.* **6**, Article 50, doi:10.1145/3534591 (2022).

11 Espino-Salinas, C. H. *et al.* Two-Dimensional Convolutional Neural Network for Depression Episodes Detection in Real Time Using Motor Activity Time Series of Depresjon Dataset. **9**, 458 (2022).

12 Frogner, J. I. *et al.* in *Proceedings of the 4th International Workshop on Multimedia for Personal Health & Health Care* 9–15 (Association for Computing Machinery, Nice, France, 2019).

13 Fukuda, S., Matsuda, Y., Tani, Y., Arakawa, Y. & Yasumoto, K. in *2020 IEEE International Conference on Pervasive Computing and Communications Workshops (PerCom Workshops).* 1-6.

14 Galván-Tejada, C. E. *et al.* Depression Episodes Detection in Unipolar and Bipolar Patients: A Methodology with Feature Extraction and Feature Selection with Genetic Algorithms Using Activity Motion Signal as Information Source. *Mobile Information Systems* **2019**, 8269695, doi:10.1155/2019/8269695 (2019).

15 Garcia-Ceja, E. *et al.* in *Proceedings of the 9th ACM Multimedia Systems Conference* 472–477 (Association for Computing Machinery, Amsterdam, Netherlands, 2018).

16 Garcia-Ceja, E. *et al.* in *2018 IEEE 31st International Symposium on Computer-Based Medical Systems (CBMS).* 316-321.

17 Ghandeharioun, A. *et al.* in *2017 Seventh International Conference on Affective Computing and Intelligent Interaction (ACII).* 325-332.

18 Griffiths, C. *et al.* Investigation of physical activity, sleep, and mental health recovery in treatment resistant depression (TRD) patients receiving repetitive transcranial magnetic stimulation (rTMS) treatment. *J Affect Disord Rep* **8**, 100337, doi:10.1016/j.jadr.2022.100337 (2022).

19 Horwitz, A. G. *et al.* Using machine learning with intensive longitudinal data to predict depression and suicidal ideation among medical interns over time. *Psychol Med*, 1-8, doi:10.1017/s0033291722003014 (2022).

20 Jacobson, N. C., Weingarden, H. & Wilhelm, S. Digital biomarkers of mood disorders and symptom change. *npj Digital Medicine* **2**, 3, doi:10.1038/s41746-019-0078-0 (2019).

21 Jakobsen, P. *et al.* Applying machine learning in motor activity time series of depressed bipolar and unipolar patients compared to healthy controls. *PLoS One* **15**, e0231995, doi:10.1371/journal.pone.0231995 (2020).
22 Jin, J. *et al.* Attention-Block Deep Learning Based Features Fusion in Wearable Social Sensor for Mental Wellbeing Evaluations. *IEEE Access* **8**, 89258-89268, doi:10.1109/ACCESS.2020.2994124 (2020).
23 Jung, D., Kim, J. & Mun, K. R. Identifying Depression in the Elderly Using Gait Accelerometry. *Annu Int Conf IEEE Eng Med Biol Soc* **2022**, 4946-4949, doi:10.1109/embc48229.2022.9871877 (2022).
24 Kim, H. *et al.* Depression Prediction by Using Ecological Momentary Assessment, Actiwatch Data, and Machine Learning: Observational Study on Older Adults Living Alone. *JMIR Mhealth Uhealth* **7**, e14149, doi:10.2196/14149 (2019).
25 Kulam, S. *Time-Series Classification with Uni-Dimensional Convolutional Neural Networks: An Experimental Comparison with Long Short-Term Memory Networks*, (2019).
26 Kumar, A., Sangwan, S. R., Arora, A. & Menon, V. G. Depress-DCNF: A deep convolutional neuro-fuzzy model for detection of depression episodes using IoMT. *Applied Soft Computing* **122**, 108863, doi:https://doi.org/10.1016/j.asoc.2022.108863 (2022).
27 Lee, H. J. *et al.* Prediction of impending mood episode recurrence using real-time digital phenotypes in major depression and bipolar disorders in South Korea: a prospective nationwide cohort study. *Psychol Med*, 1-9, doi:10.1017/s0033291722002847 (2022).
28 Llamocca, P., López, V., Santos, M. & Čukić, M. Personalized Characterization of Emotional States in Patients with Bipolar Disorder. *Mathematics* **9**, doi:10.3390/math9111174 (2021).
29 Lu, J. *et al.* Joint Modeling of Heterogeneous Sensing Data for Depression Assessment via Multi-task Learning. *Proc. ACM Interact. Mob. Wearable Ubiquitous Technol.* **2**, Article 21, doi:10.1145/3191753 (2018).
30 Mahendran, N. *et al.* Sensor-Assisted Weighted Average Ensemble Model for Detecting Major Depressive Disorder. *Sensors (Basel)* **19**, doi:10.3390/s19224822 (2019).
31 Makhmutova, M. *Predicting changes in depression using person-generated health data* Master degree thesis, Ecole polytechnique federale de Lausanne, (2021).
32 Makhmutova, M. *et al.* Predicting Changes in Depression Severity Using the PSYCHE-D (Prediction of Severity Change-Depression) Model Involving Person-Generated Health Data: Longitudinal Case-Control Observational Study. *JMIR Mhealth Uhealth* **10**, e34148, doi:10.2196/34148 (2022).
33 Mallikarjun, H. M. & Manimegalai, P. Manoglanistara - Emotional Wellness Phases Prediction of Adolescent Female Students by using Brain Waves. *Current Signal Transduction Therapy* **15**, 315-323, doi:10.2174/1574362414666190703151853 (2020).
34 Minaeva, O. *et al.* Screening for Depression in Daily Life: Development and External Validation of a Prediction Model Based on Actigraphy and Experience Sampling Method. *J Med Internet Res* **22**, e22634, doi:10.2196/22634 (2020).
35 Mullick, T., Radovic, A., Shaaban, S. & Doryab, A. Predicting Depression in Adolescents Using Mobile and Wearable Sensors: Multimodal Machine Learning-Based Exploratory Study. *JMIR Form Res* **6**, e35807, doi:10.2196/35807 (2022).
36 Narziev, N. *et al.* STDD: Short-Term Depression Detection with Passive Sensing. *Sensors (Basel)* **20**, doi:10.3390/s20051396 (2020).
37 Nguyen, D.-K., Chan, C.-L., Li, A.-H. A. & Phan, D.-V. in *2021 5th International Conference on Medical and Health Informatics* 7–12 (Association for Computing Machinery, Kyoto, Japan, 2021).

38 Nishimura, Y. *et al.* in *Sensor-and Video-Based Activity and Behavior Computing* 1-26 (Springer, 2022).
39 Asare, K. O. *et al.* Mood ratings and digital biomarkers from smartphone and wearable data differentiates and predicts depression status: A longitudinal data analysis. *Pervasive and Mobile Computing* **83**, 101621, doi:https://doi.org/10.1016/j.pmcj.2022.101621 (2022).
40 Pacheco-González, S. L. *et al.* Evaluation of Five Classifiers for Depression Episodes Detection. *Res. Comput. Sci.* **148**, 129-138, doi:10.13053/rcs-148-10-11 (2019).
41 Pedrelli, P. *et al.* Monitoring Changes in Depression Severity Using Wearable and Mobile Sensors. *Front Psychiatry* **11**, 584711, doi:10.3389/fpsyt.2020.584711 (2020).
42 Price, G. D. *et al.* An unsupervised machine learning approach using passive movement data to understand depression and schizophrenia. *J Affect Disord* **316**, 132-139, doi:10.1016/j.jad.2022.08.013 (2022).
43 Qian, K. *et al.* in *Proceedings of the Third International Symposium on Image Computing and Digital Medicine* 71–75 (Association for Computing Machinery, Xi'an, China, 2019).
44 Raihan, M., Bairagi, A. K. & Rahman, S. in *2021 12th International Conference on Computing Communication and Networking Technologies (ICCCNT).* 1-5.
45 Rodríguez-Ruiz, J. G., Galván-Tejada, C. E., Vázquez-Reyes, S., Galván-Tejada, J. I. & Gamboa-Rosales, H. Classification of Depressive Episodes Using Nighttime Data; a Multivariate and Univariate Analysis. *Program. Comput. Softw.* **46**, 689–698, doi:10.1134/s0361768820080198 (2020).
46 Rodríguez-Ruiz, J. G. *et al.* Comparison of Night, Day and 24 h Motor Activity Data for the Classification of Depressive Episodes. *Diagnostics (Basel)* **10**, doi:10.3390/diagnostics10030162 (2020).
47 Rodríguez-Ruiz, J. G. *et al.* Classification of Depressive and Schizophrenic Episodes Using Night-Time Motor Activity Signal. *Healthcare (Basel)* **10**, doi:10.3390/healthcare10071256 (2022).
48 Rykov, Y., Thach, T. Q., Bojic, I., Christopoulos, G. & Car, J. Digital Biomarkers for Depression Screening With Wearable Devices: Cross-sectional Study With Machine Learning Modeling. *JMIR Mhealth Uhealth* **9**, e24872, doi:10.2196/24872 (2021).
49 Shah, R. V. *et al.* Personalized machine learning of depressed mood using wearables. *Translational Psychiatry* **11**, 338, doi:10.1038/s41398-021-01445-0 (2021).
50 Tazawa, Y. *et al.* Evaluating depression with multimodal wristband-type wearable device: screening and assessing patient severity utilizing machine-learning. *Heliyon* **6**, e03274, doi:10.1016/j.heliyon.2020.e03274 (2020).
51 Valenza, G. *et al.* Characterization of depressive States in bipolar patients using wearable textile technology and instantaneous heart rate variability assessment. *IEEE J Biomed Health Inform* **19**, 263-274, doi:10.1109/jbhi.2014.2307584 (2015).
52 Wang, R. *et al.* Tracking Depression Dynamics in College Students Using Mobile Phone and Wearable Sensing. *Proc. ACM Interact. Mob. Wearable Ubiquitous Technol.* **2**, Article 43, doi:10.1145/3191775 (2018).
53 Xu, X. *et al.* Leveraging Routine Behavior and Contextually-Filtered Features for Depression Detection among College Students. *Proc. ACM Interact. Mob. Wearable Ubiquitous Technol.* **3**, Article 116, doi:10.1145/3351274 (2019).

54 Zanella-Calzada, L. A. *et al.* Feature Extraction in Motor Activity Signal: Towards a Depression Episodes Detection in Unipolar and Bipolar Patients. *Diagnostics (Basel)* **9**, doi:10.3390/diagnostics9010008 (2019).

Supplementary Table 2: Features of wearable AI. ANN: Artificial Neural Network; BDI-II: Beck Depression Inventory-II; BPRS: Brief Psychiatric Rating Scale; BT: Boosted Trees; CNN: Convolutional Neural Network; DAMS: Depression and Anxiety Mood Scale; DART: Dropouts Meet Multiple Additive Regression Trees; DASS: Depression Anxiety Stress Scales; DCNF: Deep convolutional neuro fuzzy; DNN: Deep Neural Network; DSM: Diagnostic and Statistical Manual of Mental Health; DT: Decision tree; ECG: Electrocardiograph; EDA: Electrodermal activity ; EEG: Electroencephalograph; ERT: Extremely Randomized Trees; GMM: Gaussian mixture models; HDRS: Hamilton Depression Rating Scale; ID3: Iterative Dichotomiser 3; KNN: K-Nearest Neighbors; LASSO: Least Absolute Shrinkage and Selection Operator; LDA: Linear discriminant analysis; LightGBM: Light Gradient Boosting Machine; LinR: Linear regression; LogR: Logistic regression; LOOCV: Leave-One-Out Cross-Validation; MADRS: Montgomery-Asberg Depression Rating Scale; MLM: Multi Level Modeling; MSE: Mean Squared Error; NB: Naive Bayes; NN: Neural Network; NPV: Negative Predictive Value; NR: Not reported; PCC: Pearson correlation coefficient ; PDSS: Panic Disorder Severity Scale; PHQ-9: Patient Health Questionnaire-9; PR: Poisson regression; QDA: Quadratic Discriminant Analysis ; QIDS: Quick Inventory of Depressive Symptomatology; r: correlation coefficient; RAE: Relative Absolute Error; RF: Random Forest; RMSE: Root Mean Square Error; RR: Ridge Regression; R-Squared: Coefficient of determination; SMAPE: Symmetric Mean Absolute Percentage Error; SVM: Support Vector Machine; VR: Voting regressor; XGBoost: extreme gradient boosting; YMRS: Young Mania Rating Scale

Study [Ref]	Name of WD	Placement of WD	Aim of AI algorithm	Problem solving approach	AI algorithm	Dataset source	Data input	Ground truth assessment	Validation approach
**Adamczyk[1]**	Actiwatch AW4	Wrist	Detection	Classification	LogR, RF, SVM	Open	Activity data	MADRS	Nested
**Ahmed[2]**	Psychorus	Wrist	Detection	Classification	CB, GB, LogR, KNN, RF, SVC, XGB	Open	Activity data, EDA data, heart rate data	BDI-II	K-fold
**Aminifar[3]**	Actiwatch AW4	Wrist	Detection	Classification	DT, ERT, ID3, RF, SVM, XGBoost	Open	Activity data	MADRS	LOOCV
**Bai[4]**	Mi Band 2	Wrist	Detection	Classification	DT, LogR, RF, SVM	Closed	Activity data, heart rate data, location, sleep data, smartphone usage data, social interaction	PHQ-9	K-fold
**Chikersal[5]**	Fitbit Flex 2	Wrist	Detection and Prediction	Classification	AdaBoost, GB, LogR, KNN, LASSO	Closed	Activity data, location, sleep data, smartphone usage data, social interaction	BDI-II	LOOCV
**Cho[6]**	Fitbit Charge HR, Fitbit Charge 2	Wrist	Prediction	Classification	RF	Closed	Activity data, heart rate data, light exposure, mood status, sleep data	DSM-5	Hold-out
**Choi[7]**	ActiGraph GT3X	Ankle, thigh, waist, wrist	Detection	Classification	LogR, MLP, SVC, XGBoost	Open	Circadian rhythms	PHQ-9	Hold-out
**Choi[8]**	Empatica E4	Wrist	Detection	Classification	DT, GB, KNN, MLP, RF, SVM, XGBoost	Closed	Activity data, EDA data, heart rate data, skin temperature	GDS, PHQ-9	K-fold
**Coutts[9]**	Biobeam	Wrist	Detection	Classification	LSTM	Closed	Heart rate data	DASS, STAI	Hold-out
**Dai[10]**	Fitbit Alta HR	Wrist	Prediction	Classification	AdaBoost, ANN, LogR, GBDT, MTL, RF, SVM	Closed	Activity data, coping, demographic data, depression level, heart rate data, negative problem orientation, post-traumatic stress disorder status, psychiatric status, sleep data	PHQ-9	K-fold
**Espino-Salinas[11]**	Actiwatch AW4	Wrist	Detection	Classification	CNN	Open	Activity data	MADRS	Hold-out, K-fold
**Frogner[12]**	Actiwatch AW4	Wrist	Detection	Classification, regression	CNN	Open	Activity data	MADRS	Hold-out, K-fold, LOOCV
**Fukuda[13]**	Fitbit Charge 3	Wrist	Detection	Classification	RF	Closed	Sleep data	DAMS	LOOCV
**Galvan-Tejada[14]**	Actiwatch AW4	Wrist	Detection	Classification	RF	Open	Activity data	MADRS	Hold-out, K-fold

**Garcia-Ceja[15]**	Actiwatch AW4	Wrist	Detection	Classification	AdaBoost, ANN, DT, KNN, GP, NB, QDA, RF, SVM, ZeroR	Closed	Activity data	MADRS	K-fold
**Garcia-Ceja[16]**	Actiwatch AW4	Wrist	Detection	Classification	DNN, RF	Open	Activity data	MADRS	LOOCV
**Ghandeharioun[17]**	Empatica E4	Wrist	Detection	Regression	AdaBoost, Ensemble model, GP, LinR, RANSAC, RF, RR	Closed	Activity data, alcohol, drug, and caffeine consumption, anxiety level, EDA data, location, mood status, sleep data, smartphone usage data, social interaction, stress level	HDRS	Hold-out, K-fold, LOOCV
**Griffiths[18]**	Fitbit	Wrist	Detection	Classification	RF	Closed	Activity data, sleep data	PHQ-9	K-fold
**Horwitz[19]**	Fitbit Charge 4	Wrist	Prediction	Classification	elasticNet, RF	Closed	Activity data, mood status, sleep data	PHQ-9	Nested
**Jacobson[20]**	Actiwatch AW4	Wrist	Detection	Classification, regression	XGBoost	Open	Activity data	MADRS	LOOCV
**Jakobsen[21]**	Actiwatch AW4	Wrist	Detection	Classification	CNN, DNN, RF	Open	Activity data	MADRS	LOOCV
**Jin[22]**	NR	Wrist	Detection	Classification	LSTM	Closed	Activity data, audio data	BDI-II, STAI	Hold-out
**Jung [23]**	Trigno Avanti Sensor	Lower back	Detection	Classification	LSTM	Closed	Activity data	DSM-IV	K-fold
**Kim[24]**	Actiwatch Spectrum PRO	Wrist	Detection	Classification	BT, DT, LogR, RF	Closed	Activity data, depression level, light exposure, sleep data	HDRS, GDS	Hold-out
**Kulam[25]**	Actiwatch AW4	Wrist	Detection	Classification	CNN, LSTM	Open	Activity data	MADRS	K-fold
**Kumar[26]**	Actiwatch AW4	Wrist	Detection	Classification	DCNF, CNN, LSTM	Open	Activity data	MADRS	Hold-out
**Lee [27]**	Fitbit Charge HR, Fitbit Charge 2, Fitbit Charge 3	Wrist	Prediction	Classification	RF	Closed	Activity data, heart rate data, light exposure, sleep data	Clinician assessment	Hold-out
**Llamocca[28]**	GENEActiv	Wrist	Detection	Classification	DT, LogR, RF, SVM	Closed	Activity data, irritibility level, motivation level, sleep data	Clinician assessment	Hold-out
**Lu[29]**	Fitbit Charge HR	Wrist	Detection	Classification, regression	LASSO, RR	Closed	Activity data, depression level, heart rate data, location, sleep data	DSM-5, QIDS	LOOCV
**Mahendran[30]**	Mi Band 3	Wrist	Detection	Classification	Ensemble model, LogR, RF	Closed	Activity data, heart rate data, sleep data	HDRS	Hold-out, K-fold
**Makhmutova[31]**	Fitbit	Wrist	Detection	Classification	Ensemble model, GBDT, LogR, RF, XGBoost	Closed	Current therapies, demographic data, depression level, health care utilization, lifestyle changes, medical history, sleep data	PHQ-9	K-fold
**Makhmutova[32]**	Fitbit	Wrist	Detection	Classification	Ensemble model	Closed	Current therapies, demographic data, depression level, health care utilization, lifestyle changes, medical history, sleep data	PHQ-9	K-fold
**Mallikarjun[33]**	MindWave Mobile	Head	Detection	Classification	CN2 Rule Inducer, NB, RF, DT, SVM	Closed	EEG data	PHQ-9	LOOCV
**Minaeva[34]**	ActiCal, GENEActiv	Wrist	Detection	Classification	LogR	Closed	Activity data, behavioural data, circadian rhythms, demographic data, emotional data, sleep data	CIDI	External validation
**Mullick[35]**	Fitbit Inspire HR	Wrist	Prediction	Regression	AdaBoost, RF, XGBoost	Closed	Activity data, heart rate data, location, sleep data, smartphone usage data, social interaction	PHQ-9	LOOCV

**Narziev**[36]	Gear S3	Wrist	Detection	Classification	RF	Closed	Activity data, light exposure, food intake, heart rate data, mood status, sleep data, smartphone use data, social interaction	BDI-II, DSM-5, PHQ-9	Hold-out, K-fold
**Nguyen**[37]	Actiwatch AW4	Wrist	Detection	Classification	CNN, Ensemble model	Open	Activity data	MADRS	Hold-out, K-fold
**Nishimura**[38]	Fitbit Charge 3	Wrist	Detection	Classification	LightGBM	Closed	Activity data, behavioural data, heart rate data, sleep data, weather data	DAMS	K-fold
**Opoku Asare**[39]	Oura Ring	Finger	Detection	Classification	KNN, LogR, RF, SVM, XGBoost	Closed	Activity data, heart rate data, location, mood status, sleep data, smartphone usage data	DASS	Nested, time-series
**Pacheco-Gonzalez**[40]	Actiwatch AW4	Wrist	Detection	Classification	DT, KNN, NB, RF, SVM	Open	Activity data	MADRS	NR
**Pedrelli**[41]	Empatica E4	Wrist	Detection	Regression	Ensemble model	Closed	Activity data, EDA data, heart rate data, location, sleep data, smartphone usage data, social interaction, weather data	HDRS	Hold-out, K-fold
**Price** [42]	Actiwatch AW4	Wrist	Detection	Classification	RF	Open	Activity data	MADRS	Nested
**Qian**[43]	Ruputer	Wrist	Detection	Classification	SVM	Closed	Activity data	NR	Hold-out
**Raihan**[44]	Actiwatch AW4	Wrist	Detection	Classification	AdaBoost, ANN, RF	Open	Activity data, demographic data	MADRS	K-fold
**Rodríguez-Ruiz**[45]	Actiwatch AW4	Wrist	Detection	Classification	RF	Open	Activity data	MADRS	Hold-out
**Rodríguez-Ruiz**[46]	Actiwatch AW4	Wrist	Detection	Classification	RF	Open	Activity data	MADRS	Hold-out
**Rodríguez-Ruiz**[47]	Actiwatch AW4	Wrist	Detection	Classification	RF	Open	Activity data	BPRS, MADRS	K-fold
**Rykov**[48]	Fitbit Charge HR, Fitbit Charge 2	Wrist	Detection	Classification, regression	XGBoost	Closed	Activity data, circadian rhythms, sleep data	PHQ-9	K-fold
**Shah**[49]	Galaxy watch	Wrist	Detection	Regression	AdaBoost, elasticNet, GB, PR, RF, SVM, VR	Closed	Activity data, anxiety level, depression level, dietary data, heart rate data, sleep data, stress level	PHQ-9	Nested
**Tazawa**[50]	Silmee W20	Wrist	Detection	Classification, regression	XGBoost	Closed	Activity data, heart rate data, skin temperature, sleep data, UV light exposure	HDRS	K-fold
**Valenza**[51]	PSYCHE	Chest	Detection	Classification	MLP	Closed	ECG data	DSM-IV, QIDS, YMRS	Hold-out, K-fold
**Wang**[52]	Microsoft Band 2	Wrist	Detection	Regression	LinR, LogR	Closed	Activity data, heart rate data, location, sleep data, smartphone usage data, social interaction	PHQ-4, PHQ-8	K-fold
**Xu**[53]	Fitbit Flex 2	Wrist	Prediction	Classification	AdaBoost	Closed	Activity data, location, sleep data, smartphone usage data, social interaction	BDI-II	External validation, Hold-out, LOOCV
**Zanella-Calzada**[54]	Actiwatch AW4	Wrist	Detection	Classification	RF	Open	Activity data	MADRS	Hold-out

1 Adamczyk, J. & Malawski, F. Comparison of Manual and Automated Feature Engineering for Daily Activity Classification in Mental Disorder Diagnosis. *Computing and Informatics* **40**, 850–879-850–879 (2021).

2 Ahmed, A. *et al.* Investigating the Feasibility of Assessing Depression Severity and Valence-Arousal with Wearable Sensors Using Discrete Wavelet Transforms and Machine Learning. *Information* **13**, 406 (2022).

3 Aminifar, A., Rabbi, F., Pun, V. K. I. & Lamo, Y. Monitoring Motor Activity Data for Detecting Patients' Depression Using Data Augmentation and Privacy-Preserving Distributed Learning. *Annu Int Conf IEEE Eng Med Biol Soc* **2021**, 2163-2169, doi:10.1109/embc46164.2021.9630592 (2021).

4 Bai, R. *et al.* Tracking and Monitoring Mood Stability of Patients With Major Depressive Disorder by Machine Learning Models Using Passive Digital Data: Prospective Naturalistic Multicenter Study. *JMIR Mhealth Uhealth* **9**, e24365, doi:10.2196/24365 (2021).

5 Chikersal, P. *et al.* Detecting Depression and Predicting its Onset Using Longitudinal Symptoms Captured by Passive Sensing: A Machine Learning Approach With Robust Feature Selection. *ACM Trans. Comput.-Hum. Interact.* **28**, Article 3, doi:10.1145/3422821 (2021).

6 Cho, C. H. *et al.* Mood Prediction of Patients With Mood Disorders by Machine Learning Using Passive Digital Phenotypes Based on the Circadian Rhythm: Prospective Observational Cohort Study. *Journal of medical Internet research* **21**, e11029, doi:10.2196/11029 (2019).

7 Choi, J. G., Ko, I. & Han, S. Depression Level Classification Using Machine Learning Classifiers Based on Actigraphy Data. *IEEE Access* **9**, 116622-116646, doi:10.1109/ACCESS.2021.3105393 (2021).

8 Choi, J. *et al.* Depressed Mood Prediction of Elderly People with a Wearable Band. *Sensors (Basel, Switzerland)* **22**, doi:10.3390/s22114174 (2022).

9 Coutts, L. V., Plans, D., Brown, A. W. & Collomosse, J. Deep learning with wearable based heart rate variability for prediction of mental and general health. *J Biomed Inform* **112**, 103610, doi:10.1016/j.jbi.2020.103610 (2020).

10 Dai, R. *et al.* Multi-Task Learning for Randomized Controlled Trials: A Case Study on Predicting Depression with Wearable Data. *Proc. ACM Interact. Mob. Wearable Ubiquitous Technol.* **6**, Article 50, doi:10.1145/3534591 (2022).

11 Espino-Salinas, C. H. *et al.* Two-Dimensional Convolutional Neural Network for Depression Episodes Detection in Real Time Using Motor Activity Time Series of Depresjon Dataset. **9**, 458 (2022).

12 Frogner, J. I. *et al.* in *Proceedings of the 4th International Workshop on Multimedia for Personal Health & Health Care* 9–15 (Association for Computing Machinery, Nice, France, 2019).

13 Fukuda, S. *et al.* in *2020 IEEE International Conference on Pervasive Computing and Communications Workshops (PerCom Workshops).* 1-6.

14 Galván-Tejada, C. E. *et al.* Depression Episodes Detection in Unipolar and Bipolar Patients: A Methodology with Feature Extraction and Feature Selection with Genetic Algorithms Using Activity Motion Signal as Information Source. *Mobile Information Systems* **2019**, 8269695, doi:10.1155/2019/8269695 (2019).

15 Garcia-Ceja, E. *et al.* in *Proceedings of the 9th ACM Multimedia Systems Conference* 472–477 (Association for Computing Machinery, Amsterdam, Netherlands, 2018).

16 Garcia-Ceja, E. *et al.* in *2018 IEEE 31st International Symposium on Computer-Based Medical Systems (CBMS).* 316-321.

17 Ghandeharioun, A. *et al.* in *2017 Seventh International Conference on Affective Computing and Intelligent Interaction (ACII).* 325-332.

18 Griffiths, C. *et al.* Investigation of physical activity, sleep, and mental health recovery in treatment resistant depression (TRD) patients receiving repetitive transcranial magnetic stimulation (rTMS) treatment. *J Affect Disord Rep* **8**, 100337, doi:10.1016/j.jadr.2022.100337 (2022).

19 Horwitz, A. G. *et al.* Using machine learning with intensive longitudinal data to predict depression and suicidal ideation among medical interns over time. *Psychol Med*, 1-8, doi:10.1017/s0033291722003014 (2022).
20 Jacobson, N. C., Weingarden, H. & Wilhelm, S. Digital biomarkers of mood disorders and symptom change. *npj Digital Medicine* **2**, 3, doi:10.1038/s41746-019-0078-0 (2019).
21 Jakobsen, P. *et al.* Applying machine learning in motor activity time series of depressed bipolar and unipolar patients compared to healthy controls. *PLoS One* **15**, e0231995, doi:10.1371/journal.pone.0231995 (2020).
22 Jin, J. *et al.* Attention-Block Deep Learning Based Features Fusion in Wearable Social Sensor for Mental Wellbeing Evaluations. *IEEE Access* **8**, 89258-89268, doi:10.1109/ACCESS.2020.2994124 (2020).
23 Jung, D., Kim, J. & Mun, K. R. Identifying Depression in the Elderly Using Gait Accelerometry. *Annu Int Conf IEEE Eng Med Biol Soc* **2022**, 4946-4949, doi:10.1109/embc48229.2022.9871877 (2022).
24 Kim, H. *et al.* Depression Prediction by Using Ecological Momentary Assessment, Actiwatch Data, and Machine Learning: Observational Study on Older Adults Living Alone. *JMIR Mhealth Uhealth* **7**, e14149, doi:10.2196/14149 (2019).
25 Kulam, S. *Time-Series Classification with Uni-Dimensional Convolutional Neural Networks: An Experimental Comparison with Long Short-Term Memory Networks*, (2019).
26 Kumar, A., Sangwan, S. R., Arora, A. & Menon, V. G. Depress-DCNF: A deep convolutional neuro-fuzzy model for detection of depression episodes using IoMT. *Applied Soft Computing* **122**, 108863, doi:https://doi.org/10.1016/j.asoc.2022.108863 (2022).
27 Lee, H. J. *et al.* Prediction of impending mood episode recurrence using real-time digital phenotypes in major depression and bipolar disorders in South Korea: a prospective nationwide cohort study. *Psychol Med*, 1-9, doi:10.1017/s0033291722002847 (2022).
28 Llamocca, P., López, V., Santos, M. & Čukić, M. Personalized Characterization of Emotional States in Patients with Bipolar Disorder. *Mathematics* **9**, doi:10.3390/math9111174 (2021).
29 Lu, J. *et al.* Joint Modeling of Heterogeneous Sensing Data for Depression Assessment via Multi-task Learning. *Proc. ACM Interact. Mob. Wearable Ubiquitous Technol.* **2**, Article 21, doi:10.1145/3191753 (2018).
30 Mahendran, N. *et al.* Sensor-Assisted Weighted Average Ensemble Model for Detecting Major Depressive Disorder. *Sensors (Basel, Switzerland)* **19**, doi:10.3390/s19224822 (2019).
31 Makhmutova, M. *Predicting changes in depression using person-generated health data* Master degree thesis, Ecole polytechnique f ed erale de Lausanne, (2021).
32 Makhmutova, M. *et al.* Predicting Changes in Depression Severity Using the PSYCHE-D (Prediction of Severity Change-Depression) Model Involving Person-Generated Health Data: Longitudinal Case-Control Observational Study. *JMIR Mhealth Uhealth* **10**, e34148, doi:10.2196/34148 (2022).
33 Mallikarjun, H. M. & Manimegalai, P. Manoglanistara - Emotional Wellness Phases Prediction of Adolescent Female Students by using Brain Waves. *Current Signal Transduction Therapy* **15**, 315-323, doi:10.2174/1574362414666190703151853 (2020).
34 Minaeva, O. *et al.* Screening for Depression in Daily Life: Development and External Validation of a Prediction Model Based on Actigraphy and Experience Sampling Method. *Journal of medical Internet research* **22**, e22634, doi:10.2196/22634 (2020).

35 Mullick, T., Radovic, A., Shaaban, S. & Doryab, A. Predicting Depression in Adolescents Using Mobile and Wearable Sensors: Multimodal Machine Learning-Based Exploratory Study. *JMIR Form Res* **6**, e35807, doi:10.2196/35807 (2022).
36 Narziev, N. *et al.* STDD: Short-Term Depression Detection with Passive Sensing. *Sensors (Basel, Switzerland)* **20**, doi:10.3390/s20051396 (2020).
37 Nguyen, D.-K., Chan, C.-L., Li, A.-H. A. & Phan, D.-V. in *2021 5th International Conference on Medical and Health Informatics* 7–12 (Association for Computing Machinery, Kyoto, Japan, 2021).
38 Nishimura, Y. *et al.* in *Sensor-and Video-Based Activity and Behavior Computing* 1-26 (Springer, 2022).
39 Asare, K. O. *et al.* Mood ratings and digital biomarkers from smartphone and wearable data differentiates and predicts depression status: A longitudinal data analysis. *Pervasive and Mobile Computing* **83**, 101621, doi:https://doi.org/10.1016/j.pmcj.2022.101621 (2022).
40 Pacheco-González, S. L. *et al.* Evaluation of Five Classifiers for Depression Episodes Detection. *Res. Comput. Sci.* **148**, 129-138, doi:10.13053/rcs-148-10-11 (2019).
41 Pedrelli, P. *et al.* Monitoring Changes in Depression Severity Using Wearable and Mobile Sensors. *Frontiers in psychiatry* **11**, 584711, doi:10.3389/fpsyt.2020.584711 (2020).
42 Price, G. D. *et al.* An unsupervised machine learning approach using passive movement data to understand depression and schizophrenia. *Journal of affective disorders* **316**, 132-139, doi:10.1016/j.jad.2022.08.013 (2022).
43 Qian, K. *et al.* in *Proceedings of the Third International Symposium on Image Computing and Digital Medicine* 71–75 (Association for Computing Machinery, Xi'an, China, 2019).
44 Raihan, M., Bairagi, A. K. & Rahman, S. in *2021 12th International Conference on Computing Communication and Networking Technologies (ICCCNT).* 1-5.
45 Rodríguez-Ruiz, J. G. *et al.* Classification of Depressive Episodes Using Nighttime Data; a Multivariate and Univariate Analysis. *Program. Comput. Softw.* **46**, 689–698, doi:10.1134/s0361768820080198 (2020).
46 Rodríguez-Ruiz, J. G. *et al.* Comparison of Night, Day and 24 h Motor Activity Data for the Classification of Depressive Episodes. *Diagnostics (Basel)* **10**, doi:10.3390/diagnostics10030162 (2020).
47 Rodríguez-Ruiz, J. G. *et al.* Classification of Depressive and Schizophrenic Episodes Using Night-Time Motor Activity Signal. *Healthcare (Basel)* **10**, doi:10.3390/healthcare10071256 (2022).
48 Rykov, Y. *et al.* Digital Biomarkers for Depression Screening With Wearable Devices: Cross-sectional Study With Machine Learning Modeling. *JMIR Mhealth Uhealth* **9**, e24872, doi:10.2196/24872 (2021).
49 Shah, R. V. *et al.* Personalized machine learning of depressed mood using wearables. *Translational Psychiatry* **11**, 338, doi:10.1038/s41398-021-01445-0 (2021).
50 Tazawa, Y. *et al.* Evaluating depression with multimodal wristband-type wearable device: screening and assessing patient severity utilizing machine-learning. *Heliyon* **6**, e03274, doi:10.1016/j.heliyon.2020.e03274 (2020).
51 Valenza, G. *et al.* Characterization of depressive States in bipolar patients using wearable textile technology and instantaneous heart rate variability assessment. *IEEE J Biomed Health Inform* **19**, 263-274, doi:10.1109/jbhi.2014.2307584 (2015).

52 Wang, R. *et al.* Tracking Depression Dynamics in College Students Using Mobile Phone and Wearable Sensing. *Proc. ACM Interact. Mob. Wearable Ubiquitous Technol.* **2**, Article 43, doi:10.1145/3191775 (2018).
53 Xu, X. *et al.* Leveraging Routine Behavior and Contextually-Filtered Features for Depression Detection among College Students. *Proc. ACM Interact. Mob. Wearable Ubiquitous Technol.* **3**, Article 116, doi:10.1145/3351274 (2019).
54 Zanella-Calzada, L. A. *et al.* Feature Extraction in Motor Activity Signal: Towards a Depression Episodes Detection in Unipolar and Bipolar Patients. *Diagnostics (Basel)* **9**, doi:10.3390/diagnostics9010008 (2019).

Supplementary Table 3: Reviewers' judgments about each domain in "risk of bias" and "applicability concerns" for each included study. D: Domain; Plus inside a green circle: low risk, Question mark inside yellow circle: Some concerns; Minus inside red circle: high risk.

	Risk of Bias				Applicability Concerns		
**Study ID**	**D1**	**D2**	**D3**	**D4**	**D1**	**D2**	**D3**
Adamczyk 2021	+	+	+	+	+	+	+
Ahmed 2022	+	+	+	+	+	+	+
Aminifar 2021	?	-	+	+	+	+	+
Bai 2021	+	?	+	-	+	+	+
Chikersal 2021	+	+	+	+	+	+	+
Cho 2019	?	+	+	+	+	+	+
Choi 2021a	+	+	+	+	+	+	+
Choi 2022b	-	+	?	-	-	+	+
Coutts 2020	+	+	+	-	+	+	+
Dai 2022	-	+	+	+	+	+	+
Espino-Salinas 2022	-	+	+	+	+	+	+
Frogner 2019	?	+	+	-	+	+	+
Fukuda 2020	+	+	+	+	+	+	+
Galvan-Tejada 2019	+	+	+	+	+	+	+
Garcia-Ceja 2018a	+	+	+	+	+	+	+
Garcia-Ceja 2018b	+	+	+	+	+	+	+
Ghandeharioun 2017	-	+	-	+	+	+	+
Griffiths 2022	-	+	+	+	+	+	+
Horwitz 2022	?	+	+	-	+	+	+
Jacobson 2019	-	+	+	+	+	+	+
Jakobsen 2020	+	+	+	+	+	+	+
Jin 2020	-	-	+	-	+	+	+
Jung 2022	-	+	?	+	+	+	+
Kim 2019	-	+	+	-	+	+	+
Kulam 2019	+	+	+	+	+	+	+
Kumar 2022	-	+	+	+	+	+	+
Lee 2022	+	+	+	+	+	+	+

+	Low risk
?	Some concerns
-	High risk

D1	Participant
D2	Index test
D3	Reference Standard
D4	Analysis

Llamocca 2021	?	+	+	-	?	+	+
Lu 2018	?	+	+	+	?	+	+
Mahendran 2019	?	-	+	+	?	+	+
Makhmutova 2021	?	-	+	+	+	+	+
Makhmutova 2022	?	-	+	+	+	+	+
Mallikarjun 2020	-	+	+	-	+	+	+
Minaeva 2020	+	+	+	+	+	+	+
Mullick 2022	-	+	+	+	+	+	+
Narziev 2020	-	-	-	+	-	+	+
Nguyen 2021	-	+	+	+	+	+	+
Nishimura 2022	?	+	+	+	+	+	+
Opoku Asare 2022	-	+	-	+	-	+	+
Pacheco-Gonzalez 2019	?	+	+	?	+	+	+
Pedrelli 2020	?	+	+	+	+	+	+
Price 2022	+	+	+	+	+	+	+
Qian 2019	-	+	?	+	-	+	?
Raihan 2021	-	+	+	+	+	+	+
Rodríguez-Ruiz 2020a	-	+	+	-	+	+	+
Rodríguez-Ruiz 2020b	-	+	+	-	+	+	+
Rodríguez-Ruiz 2022	+	+	+	+	+	+	+
Rykov 2021	-	+	+	+	+	+	+
Shah 2021	-	+	+	+	+	+	+
Tazawa 2020	+	+	+	+	+	+	+
Valenza 2015	-	+	+	+	+	+	+
Wang 2018	?	+	+	+	+	+	+
Xu 2019	+	+	+	+	+	+	+
Zanella-Calzada 2019	-	+	+	+	+	+	+

Supplementary Table 4: Pooled mean estimates of highest accuracy by several factors. BDI-II: Beck Depression Inventory-II; CI: Confidence interval; DSM: Diagnostic and Statistical Manual of Mental Health; HDRS: Hamilton Depression Rating Scale; MADRS: Montgomery-Asberg Depression Rating Scale; NA: Not applicable; PHQ-9: Patient Health Questionnaire-9; WD: Wearable device

Groups	Number of studies	Sample size	Accuracy (%)	Pooled mean accuracy	Heterogeneity measures			Test for subgroup differences
	Total n	Total N	Range	Mean (%) (95% CI)	Tau^2	Q (p-value)	I^2 (%)	Q (p-value)
**Algorithms**								
Random forest	19	105,716	0.64-1.00	0.85 (0.79-0.90)	0.04	5119.9 (<0.001)	99.6	37.84 (<0.001)
Logistic regression	8	21,353	0.56-0.93	0.76 (0.68-0.84)		603.7 (<0.001)	98.8	
XGBoost	7	28,116	0.61-0.97	0.88 (0.82-0.94)		2712.7 (<0.001)	99.8	
Convolutional neural network	5	6,131	0.76-1.00	0.85 (0.74-0.92)		374.9 (<0.001)	98.9	
Support vector machine	4	6,181	0.66-0.78	0.79 (0.69-0.88)		8.9 (0.081)	66.2	
Ensemble model	4	22,996	0.68-0.99	0.86 (0.78-0.93)		1272.0 (<0.001)	99.8	
Decision tree	3	5,696	0.66-0.93	0.79 (0.68-0.88)		46.8 (<0.001)	95.7	
Long short-term memory	3	1,337	0.73-0.96	0.85 (0.71-0.95)		111.5 (<0.001)	98.2	
AdaBoost	2	139	0.88-0.98	0.99 (0.92-1.00)		6.3 (0.173)	84.0	
Deep neural network	2	104	0.71-0.84	0.86 (0.72-0.96)		2.5 (0.321)	59.2	
K-nearest neighbors	2	2,141	0.58-0.66	0.81 (0.70-0.91)		2.9 (0.246)	65.7	
Gradient boosting	2	2,141	0.64-0.69	0.85 (0.74-0.93)		1.2 (0.548)	15.9	
Multilayer perceptron	2	11,291	0.84-1.00	0.85 (0.73-0.94)		1335.8 (<0.001)	99.9	
Support vector classifier	2	11,192	0.60-0.85	0.83 (0.73-0.91)		524.0 (<0.001)	99.8	
**Aims of AI**								
Detection	73	175,215	0.56-1.00	0.89 (0.82-0.93)	2.6	8623.9 (<.001)	99.2	0.33 (0.567)
Prediction	2	73,988	0.67-0.90	0.81 (0.30-0.98)		345.7 (<.001)	99.7	
**Wearable devices**								
Actiwatch	31	53,617	0.65-1.00	0.91 (0.83-0.96)	2.5	2083.8 (<0.001)	98.6	2.17 (0.34)
Fitbit	16	140,123	0.63-0.92	0.80 (0.57-0.92)		4791.6 (<0.001)	99.7	
Mi Band 2	7	1,813	0.75-0.99	0.91 (0.55-0.99)		115. 9 (<0.001)	94.8	
**Data sources**								
WD-based	40	72,137	0.56-1.00	0.91 (0.83-0.95)	2.8	2986.5 (<0.001)	98.6	1.05 (0.592)
WD-based & self-reported	22	102,610	0.67-0.99	0.86 (0.67-0.95)		3738.9 (<0.001)	99.4	
WD-based & non-WD based	10	74,356	0.63-0.90	0.81 (0.40-0.96)		312.5 (<0.001)	97.1	
**Data types**								
Activity data	30	54,338	0.65-1.00	0.91 (0.83-0.96)	2.6	2159.8 (<0.001)	98.7	1.07 (0.587)
Activity data & others	28	91,393	0.56-0.99	0.85 (0.68-0.93)		7346.5 (<0.001)	99.6	
Non-activity data	17	103,472	0.64-1.00	0.88 (0.70-0.96)		3601.7 (<0.001)	99.6	
**Reference standards**								

MADRS	30	52,312	0.65-1.00	0.91 (0.80-0.96)		1856.7 (<0.001)	98.4	
PHQ-9	19	99,948	0.68-0.97	0.84 (0.58-0.95)		3483.9 (<0.001)	99.5	
BDI-II	12	14,750	0.56-0.88	0.66 (0.16-0.95)	3.0	79.9 (<0.001)	86.2	3.60 (0.462)
HDRS	4	1,586	0.76-0.99	0.92 (0.51-0.99)		100.4 (<0.001)	97.0	
DSM	3	4,635	0.67-1.00	0.97 (0.80-1.00)		250.0 (<0.001)	99.2	
**All studies**	**75**	**249,203**	**0.56-1.00**	**0.89 (0.83-0.93)**	**2.55**	**14657.1 (<0.001)**	**99.5**	**NA**

Supplementary Table 5: Pooled mean estimates of lowest accuracy by several factors. BDI-II: Beck Depression Inventory-II; CI: Confidence interval; DSM: Diagnostic and Statistical Manual of Mental Health; MADRS: Montgomery-Asberg Depression Rating Scale; NA: Not applicable; PHQ-9: Patient Health Questionnaire-9; WD: Wearable device

Groups	Number of studies	Sample size	Accuracy (%)	Pooled mean accuracy	Heterogeneity measures			Test for subgroup differences
	Total n	Total N	Range	Mean (%) (95% CI)	Tau^2	Q (p-value)	I^2 (%)	Q (p-value)
**Algorithms**								
Random forest	9	9,094	0.61-0.99	0.64 (0.55-0.72)	0.03	2231.2 (<0.001)	99.6	96.55 (<0.001)
Convolutional neural network	4	1,040	0.71-0.77	0.70 (0.59-0.81)		1.3 (0.535)	0.0	
Logistic regression	4	1,291	0.29-0.70	0.63 (0.54-0.72)		177.2 (<0.001)	98.3	
Support vector machine	3	681	0.59-0.75	0.67 (0.57-0.78)		15.4 (<0.001)	87.0	
XGBoost	3	1,321	0.20-0.74	0.55 (0.45-0.64)		317.2 (<0.001)	99.4	
Ensemble model	3	22,546	0.62-0.86	0.78 (0.67-0.90)		330.4 (<0.001)	99.4	
Multilayer perceptron	2	20,83	0.33-0.65	0.67 (0.58-0.77)		363.6 (<0.001)	99.7	
Deep neural network	2	110	0.65-0.67	0.65 (0.52-0.78)		0.00 (0.980)	0.0	
AdaBoost	2	157	0.76-0.97	0.96 (0.83-1.00)		17.5 (<0.001)	94.3	
**Wearable devices**								
Actiwatch	18	10,451	0.62-0.99	0.80 (0.70-0.87)	0.9	907.9 (<0.001)	98.1	3.71 (0.054)
Fitbit	8	22,816	0.45-0.86	0.63 (0.45-0.77)		134.4 (<.0001)	94.8	
**Data sources**								
WD-based	18	14,579	0.54-0.99	0.72 (0.60-0.81)	1.0	1306.4 (<0.001)	98.7	0.84 (0.657)
WD-based & self-reported	14	26,766	0.20-0.97	0.64 (0.46-0.78)		1556.9 (<0.001)	99.2	
WD-based & non-WD based	6	879	0.45-0.76	0.64 (0.33-0.87)		42.5 (<0.001)	88.2	
**Data types**								
Activity data	16	10,731	0.59-0.99	0.75 (0.62-0.84)	0.9	1011.4 (<0.001)	98.5	2.55 (0.279)
Activity data & others	15	4,194	0.45-0.97	0.70 (0.55-0.81)		168.2 (<0.001)	91.7	
Non-activity data	8	27,399	0.20-0.72	0.57 (0.36-0.75)		1551.5 (<0.001)	99.5	
**Reference standards**								
MADRS	17	9,138	0.62-0.99	0.80 (0.68-0.88)	0.9	849.9 (<0.001)	98.1	6.82 (0.078)
PHQ-9	10	26,402	0.20-0.75	0.56 (0.37-0.74)		1528.4 (<0.001)	99.4	
BDI-II	4	2,327	0.45-0.76	0.56 (0.26-0.82)		18.6 (0.064)	83.8	
DSM	2	1,690	0.61-0.65	0.55 (0.24-0.82)		91.8 (<0.001)	98.9	
**All studies**	**39**	**44,846**	**0.20-0.99**	**0.70 (0.62-0.78)**	**0.9**	**3322.7 (<0.001)**	**98.9**	**NA**

Supplementary Table 6: Pooled mean estimates of highest sensitivity by several factors. BDI-II: Beck Depression Inventory-II; CI: Confidence interval; DSM: Diagnostic and Statistical Manual of Mental Health; MADRS: Montgomery-Asberg Depression Rating Scale; NA: Not applicable; PHQ-9: Patient Health Questionnaire-9; WD: Wearable device

Groups	Number of studies	Sample size	Sensitivity (%)	Pooled mean sensitivity	Heterogeneity measures			Test for subgroup differences
	Total n	Total N	Range	Mean (%) (95% CI)	Tau^2	Q (p-value)	I^2 (%)	Q (p-value)
**Algorithms**								
Random forest	17	19,116	0.61-0.99	0.79 (0.73-0.85)	0.02	2971.1 (<0.001)	99.5	24.05 (0.002)
XGBoost	6	5,799	0.65-0.91	0.80 (0.74-0.87)		350.2 (<0.001)	98.6	
Logistic regression	5	3,435	0.60-0.90	0.79 (0.72-0.86)		149.7 (<0.001)	97.3	
Convolutional neural network	4	2,022	0.65-0.99	0.83 (0.73-0.94)		51.4 (<0.001)	94.2	
Support vector machine	4	2,548	0.53-0.89	0.74 (0.66-0.82)		64.0 (<0.001)	95.3	
Ensemble model	3	4,771	0.55-0.92	0.88 (0.80-0.96)		750.0 (<0.001)	99.7	
Decision tree	3	2,400	0.74-0.92	0.70 (0.62-0.78)		10.3 (<0.001)	80.5	
AdaBoost	2	56	0.82-1.00	0.96 (0.82-1.00)		6.2 (0.046)	83.8	
Deep neural network	2	40	0.57-0.82	0.78 (0.60-0.96)		3.5 (0.176)	71.2	
**Aims of AI**								
Detection	56	45,943	0.53-1.00	0.88 (0.80-0.93)	2.2	2730.6 (<0.001)	98.0	0.71 (0.400)
Prediction	2	8,226	0.61-0.83	0.74 (0.27-0.96)		19.4 (<0.001)	94.8	
**Wearable devices**								
Actiwatch	28	21,328	0.57-1.00	0. 90 (0.81-0.95)	1.8	754.6 (<0.001)	96.4	2.44 (0.119)
Fitbit	10	21,809	0.55-0.88	0.75 (0.51-0.90)		959.2 (<0.001)	99.1	
**Data sources**								
WD-based	37	31,193	0.53-1.00	0.90 (0.82-0.95)	2.2	1889.9 (<0.001)	98.1	2.20 (0.332)
WD-based & self-reported	15	14,456	0.55-1.00	0.77 (0.53-0.91)		819.7 (<0.001)	98.3	
WD-based & non-WD based	6	8,520	0.74-0.90	0.84 (0.49-0.97)		11.5 (0.083)	56.6	
**Data types**								
Activity data	27	21,608	0.53-0.99	0.89 (0.80-0.94)	2.2	859.9 (<0.001)	97.0	1.65 (0.437)
Activity data & others	20	16,370	0.60-1.00	0.80 (0.59-0.91)		761.7 (<0.001)	97.5	
Non-activity data	11	16,191	0.55-1.00	0.91 (0.68-0.98)		809.9 (<0.001)	98.8	
**Reference standards**								
MADRS	27	20,870	0.57-1.00	0.89 (0.77-0.95)	2.6	712.6 (<0.001)	96.4	3.04 (0.385)
PHQ-9	15	14,028	0.55-0.92	0.80 (0.50-0.94)		810.1 (<0.001)	98.3	
BDI-II	8	7,551	0.60-0.82	0.74 (0.23-0.96)		37.4 (<0.001)	81.3	
DSM	3	2,800	0.61-1.00	0.96 (0.79-1.00)		62.9 (<0.001)	96.8	
**All studies**	**58**	**54,169**	**0.53-1.00**	**0.87 (0.79-0.92)**	**2.1**	**2925.3 (<0.001)**	**98.1**	**NA**

Supplementary Table 7: Pooled mean estimates of lowest sensitivity by several factors. BDI-II: Beck Depression Inventory-II; CI: Confidence interval; DSM: Diagnostic and Statistical Manual of Mental Health; MADRS: Montgomery-Asberg Depression Rating Scale; NA: Not applicable; PHQ-9: Patient Health Questionnaire-9; WD: Wearable device

Groups	Number of studies	Sample size	Sensitivity (%)	Pooled mean sensitivity	Heterogeneity measures			Test for subgroup differences
	Total n	Total N	Range	Mean (%) (95% CI)	Tau²	Q (p-value)	I² (%)	Q (p-value)
**Algorithms**								
Random forest	9	3,628	0.39-0.98	0.58 (0.39-0.76)	1.7	339.9 (<0.001)	97.6	2.56 (0.862)
Convolutional neural network	4	360	0.53-0.66	0.60 (0.35-0.80)		2.9 (0.574)	0.0	
Logistic regression	3	155	0.46-0.84	0.69 (0.42-0.87)		12.8 (0.005)	84.4	
Ensemble model	3	4,771	0.20-0.77	0.61 (0.33-0.84)		637.8 (<0.001)	99.7	
Support vector machine	3	248	0.29-0.84	0.63 (0.36-0.84)		42.4 (<0.001)	95.3	
XGBoost	2	131	0.00-0.66	0.32 (0.05-0.80)		9.6 (0.008)	89.6	
Deep neural network	2	46	0.43-0.43	0.48 (0.22-0.76)		0.00 (1.00)	0.0	
**Wearable devices**								
Actiwatch	17	4,095	0.43-0.98	0.69 (0.52-0.82)	1.4	329.3 (<0.001)	95.1	4.32 (0.038)
Fitbit	5	4,711	0.00-0.66	0.35 (0.15-0.62)		524.7 (<0.001)	99.2	
**Data sources**								
WD-based	17	7,853	0.00-0.98	0.64 (0.48-0.77)	1.3	527.7 (<0.001)	97.0	1.47 (0.480)
WD-based & self-reported	9	4,823	0.20-0.91	0.51 (0.29-0.72)		554.0 (<0.001)	98.6	
WD-based & non-WD based	4	339	0.66-0.84	0.73 (0.37-0.93)		13.1 (0.001)	77.1	
**Data types**								
Activity data	16	4,190	0.29-0.98	0.64 (0.47-0.79)	1.4	412.8 (<0.001)	96.4	0.56 (0.755)
Activity data & others	11	1,794	0.00-0.91	0.61 (0.40-0.78)		88.6 (<0.001)	88.7	
Non-activity data	3	7,031	0.20-0.79	0.50 (0.21-0.79)		1413.3 (<0.001)	99.9	
**Reference standards**								
MADRS	16	3,631	0.43-0.98	0.64 (0.49-0.79)	0.05	913.0 (<0.001)	98.4	3.36 (0.340)
PHQ-9	6	4,836	0.00-0.84	0.39 (0.17-0.62)		1125.8 (<0.001)	99.6	
BDI-II	2	1,103	0.63-0.66	0.64 (0.31-0.97)		0.1 (0.945)	0.0	
DSM	2	1,620	0.25-0.42	0.59 (0.27-0.92)		97.5 (<0.001)	99.0	
**All studies**	**30**	**13,015**	**0.00-0.98**	**0.61 (0.49-0.72)**	**1.3**	**2109.6 (<0.001)**	**98.6**	**NA**

Supplementary Table 8: Pooled mean estimates of highest specificity by several factors. BDI-II: Beck Depression Inventory-II; CI: Confidence interval; DSM: Diagnostic and Statistical Manual of Mental Health; MADRS: Montgomery-Asberg Depression Rating Scale; NA: Not applicable; PHQ-9: Patient Health Questionnaire-9; WD: Wearable device

Groups	Number of studies	Sample size	Specificity (%)	Pooled mean specificity	Heterogeneity measures			Test for subgroup differences
	Total n	Total N	Range	Mean (%) (95% CI)	Tau^2	Q (p-value)	I^2 (%)	Q (p-value)
**Algorithms**								
Random forest	16	86,012	0.60-1.00	0.86 (0.80-0.92)	3.6	7784.3 (<0.001)	99.8	16.02 (0.042)
XGBoost	6	13,150	0.56-1.00	0.86 (0.79-0.94)		1977.2 (<0.001)	99.7	
Logistic regression	4	9,632	0.51-1.00	0.80 (0.72-0.87)		325.2 (<0.001)	99.1	
Convolutional neural network	4	3,775	0.81-1.00	0.86 (0.76-0.97)		24.8 (<0.001)	87.9	
Support vector machine	3	3,559	0.60-0.94	0.79 (0.71-0.88)		149.8 (<0.001)	98.7	
Ensemble model	3	17,775	0.80-0.98	0.84 (0.75-0.93)		758.7 (<0.001)	99.7	
Decision tree	2	3,248	0.57-0.94	0.78 (0.69-0.87)		154.3 (<0.001)	99.4	
AdaBoost	2	86	0.96-0.97	1.00 (0.87-1.00)		0.02 (0.990)	0.0	
Deep neural network	2	64	0.84-0.91	0.92 (0.78-1.00)		0.57 (0.704)	0.0	
**Aims of AI**								
Detection	52	91,905	0.51-1.00	0.94 (0.88-0.97)	3.5	5572.4 (<0.001)	99.1	0.57 (0.452)
Prediction	2	65,671	0.74-0.91	0.84 (0.29-0.99)		139.0 (<0.001)	99.3	
**Wearable devices**								
Actiwatch	28	31,265	0.56-1.00	0.95 (0.88-0.98)	3.9	1287.7 (<0.001)	97.9	0.35 (0.556)
Fitbit	10	117,634	0.74-1.00	0.92 (0.68-0.98)		2603.1 (<0.001)	99.7	
**Data sources**								
WD-based	37	39,267	0.51-1.00	0.95 (0.89-0.98)	3.5	1482.6 (<0.001)	97.6	1.57 (0.456)
WD-based & self-reported	15	53,035	0.69-1.00	0.87 (0.61-0.96)		541.2 (<0.001)	97.4	
WD-based & non-WD based	2	65,274	0.91-0.96	0.94 (0.52-1.00)		1.7 (0.433)	40.2	
**Data types**								
Activity data	27	31,706	0.56-1.00	0.95 (0.88-0.98)	3.5	1360.1 (<0.001)	98.1	1.11 (0.573)
Activity data & others	16	72,911	0.51-1.00	0.89 (0.68-0.97)		5396.5 (<0.001)	99.7	
Non-activity data	11	52,959	0.67-1.00	0.92 (0.65-0.99)		539.2 (<0.001)	98.1	
**Reference standards**								
MADRS	27	30,418	0.56-1.00	0.95 (0.85-0.98)	4.5	1118.0 (<0.001)	97.7	0.73 (0.866)
PHQ-9	11	52,101	0.67-1.00	0.92 (0.56-0.99)		41.1 (<0.001)	83.0	
BDI-II	8	6,774	0.51-0.96	0.84 (0.21-0.99)		475.4 (<0.001)	97.9	
DSM	3	1,744	0.74-1.00	0.96 (0.66-1.00)		113.3 (<0.001)	98.2	
**All studies**	**54**	**157,576**	**0.51-1.00**	**0.93 (0.87-0.97)**	**3.4**	**11879.1 (<0.001)**	**99.6**	**NA**

Supplementary Table 9: Pooled mean estimates of lowest specificity by several factors. BDI-II: Beck Depression Inventory-II; CI: Confidence interval; MADRS: Montgomery-Asberg Depression Rating Scale; NA: Not applicable; PHQ-9: Patient Health Questionnaire-9; WD: Wearable device.

Groups	Number of studies	Sample size	Specificity (%)	Pooled mean specificity	Heterogeneity measures			Test for subgroup differences
	Total n	Total N	Range	Mean (%) (95% CI)	Tau^2	Q (p-value)	I^2 (%)	Q (p-value)
**Algorithms**								
Random forest	8	5,433	0.42-0.99	0.70 (0.60-0.80)	0.03	1422.2 (<0.001)	99.5	27.23 (<0.001)
Convolutional neural network	4	679	0.78-0.82	0.72 (0.60-0.84)		1.1 (0.902)	0.0	
Ensemble model	3	17,775	0.65-0.90	0.80 (0.68-0.92)		352.4 (<0.001)	99.4	
XGBoost	2	166	0.57-0.79	0.69 (0.45-0.93)		6.7 (0.035)	85.0	
Logistic regression	2	58	0.62-0.74	0.61 (0.44-0.79)		1.2 (0.548)	17.4	
Support vector machine	2	391	0.62-0.87	0.72 (0.57-0.88)		15.6 (<0.001)	93.6	
AdaBoost	2	82	0.82-0.94	0.98 (0.81-1.00)		2.9 (0.235)	65.4	
Deep neural network	2	64	0.69-0.78	0.69 (0.53-0.84)		0.73 (0.947)	0.0	
**Wearable devices**								
Actiwatch	17	6,277	0.59-0.99	0.84 (0.74-0.90)	0.9	699.9 (<0.001)	97.7	4.64 (0.031)
Fitbit	5	17,862	0.42-0.82	0.63 (0.42-0.80)		120.2 (<0.001)	96.7	
**Data sources**								
WD-based	17	8,648	0.25-0.99	0.75 (0.62-0.85)	1.4	1745.2 (<0.001)	99.1	0.57 (0.449)
WD-based & self-reported	9	17,956	0.42-0.94	0.66 (0.43-0.83)		134.9 (<0.001)	94.1	
**Data types**								
Activity data	16	6,540	0.60-0.99	0.75 (0.73-0.90)	1.0	934.1 (<0.001)	98.9	16.72 (0.083)
Activity data & others	8	1,808	0.40-0.94	0.68 (0.45-0.79)		104.5 (<0.001)	93.3	
Non-activity data	3	18,306	0.25-0.65	0.56 (0.26-0.76)		562.5 (<0.001)	99.6	
**Reference standards**								
MADRS	16	5,428	0.59-0.99	0.77 (0.73-0.86)	0.04	902.0 (<0.001)	98.3	19.17 (0.101)
PHQ-9	3	17,270	0.57-0.65	0.65 (0.49-0.77)		2.2 (0.512)	10.9	
BDI-II	2	1,010	0.40-0.82	0.61 (0.42-0.76)		54.8 (<0.001)	98.2	
**All studies**	**27**	**26,654**	**0.25-0.99**	**0.73 (0.62-0.82)**	**1.3**	**1909.2 (<0.001)**	**98.6**	**NA**

Supplementary Table 10: PRISMA-DTA Checklist. DTA: diagnostic test accuracy

Section/topic	#	PRISMA-DTA Checklist Item	Reported on page #
**TITLE / ABSTRACT**			
Title	1	Identify the report as a systematic review (+/- meta-analysis) of diagnostic test accuracy (DTA) studies.	1
Abstract	2	Abstract: See PRISMA-DTA for abstracts.	2
**INTRODUCTION**			
Rationale	3	Describe the rationale for the review in the context of what is already known.	4
Clinical role of index test	D1	State the scientific and clinical background, including the intended use and clinical role of the index test, and if applicable, the rationale for minimally acceptable test accuracy (or minimum difference in accuracy for comparative design).	3
Objectives	4	Provide an explicit statement of question(s) being addressed in terms of participants, index test(s), and target condition(s).	4
**METHODS**			
Protocol and registration	5	Indicate if a review protocol exists, if and where it can be accessed (e.g., Web address), and, if available, provide registration information including registration number.	5
Eligibility criteria	6	Specify study characteristics (participants, setting, index test(s), reference standard(s), target condition(s), and study design) and report characteristics (e.g., years considered, language, publication status) used as criteria for eligibility, giving rationale.	6
Information sources	7	Describe all information sources (e.g., databases with dates of coverage, contact with study authors to identify additional studies) in the search and date last searched.	5
Search	8	Present full search strategies for all electronic databases and other sources searched, including any limits used, such that they could be repeated.	5
Study selection	9	State the process for selecting studies (i.e., screening, eligibility, included in systematic review, and, if applicable, included in the meta-analysis).	6
Data collection process	10	Describe method of data extraction from reports (e.g., piloted forms, independently, in duplicate) and any processes for obtaining and confirming data from investigators.	6
Definitions for data extraction	11	Provide definitions used in data extraction and classifications of target condition(s), index test(s), reference standard(s) and other characteristics (e.g. study design, clinical setting).	7
Risk of bias and applicability	12	Describe methods used for assessing risk of bias in individual studies and concerns regarding the applicability to the review question.	7
Diagnostic accuracy	13	State the principal diagnostic accuracy measure(s) reported (e.g. sensitivity, specificity) and state the	7

measures		unit of assessment (e.g. per-patient, per-lesion).	
Synthesis of results	14	Describe methods of handling data, combining results of studies and describing variability between studies. This could include, but is not limited to: a) handling of multiple definitions of target condition. b) handling of multiple thresholds of test positivity, c) handling multiple index test readers, d) handling of indeterminate test results, e) grouping and comparing tests, f) handling of different reference standards	8

Supplementary Table 11: Search strategy

Database(s): **Ovid MEDLINE(R) ALL** 1946 to October 03, 2022.

#	Searches	Results
1	exp Artificial Intelligence/	147056
2	"Artificial Intelligence".tw.	19111
3	exp Machine Learning/	45132
4	"Machine Learning".tw.	55796
5	exp Deep Learning/	11216
6	"Deep Learning".tw.	26682
7	"supervised learning".tw.	3641
8	"unsupervised learning".tw.	1715
9	"reinforcement learning".tw.	4214
10	"Decision tree".tw.	10101
11	"K-Nearest Neighbor*".tw.	3833
12	"Support vector machine*".tw.	20729
13	"Recurrent neural network*".tw.	3179
14	"convolutional neural network*".tw.	15429
15	"Artificial neural network*".tw.	14226
16	"Deep Neural Networks".tw.	3037
17	"Naïve Bayes".tw.	3
18	"Naive Bayes".tw.	2376
19	"Fuzzy Logic".tw.	2155
20	"K-Means".tw.	5574
21	"Random Forest".tw.	13210
22	"Long Short-Term Memory Networks".tw.	138
23	autoencoder.tw.	1529
24	"boltzmann machine".tw.	262
25	"deep belief network".tw.	252
26	"Gradient Boost*".tw.	3008
27	AdaBoost.tw.	963
28	"Multilayer Perceptron".tw.	1939
29	"Ensemble learning".tw.	1147
30	exp Wearable Electronic Devices/	16804
31	wearable*.tw.	19321
32	"smart watch*".tw.	182
33	smartwatch*.tw.	669
34	acceleromet*.tw.	19744
35	gyroscop*.tw.	2138
36	"inertial sensor".tw.	994
37	"inertial measurement unit*".tw.	2192
38	"fitness band*".tw.	20
39	"flexible band*".tw.	87
40	headband*.tw.	310
41	"head band*".tw.	72
42	wristband*.tw.	630
43	"smart insole*".tw.	40
44	"Smart armband".tw.	1
45	bracelet*.tw.	599
46	Emotiv.tw.	80
47	NeuroSky.tw.	15

48	Mindo.tw.	44
49	StarLab.tw.	2
50	EmSense.tw.	0
51	"B-Alert X24".tw.	0
52	Enobio.tw.	6
53	BrainBit.tw.	0
54	NeuroSky.tw.	15
55	Muse.tw.	568
56	OpenBCI.tw.	14
57	Neuroelectrics.tw.	2
58	"G.tec nautilus".tw.	0
59	BioSemi.tw.	24
60	"mBrainTrain".tw.	2
61	Cognionics.tw.	4
62	"CGX QUICK".tw.	0
63	Fitbit.tw.	934
64	Garmin.tw.	213
65	"Misfit shine".tw.	23
66	"Polar loop".tw.	31
67	Jawbone.tw.	804
68	Geneactiv.tw.	155
69	Empatica.tw.	71
70	Amiigo.tw.	0
71	Actigraph.tw.	3259
72	"Apple Watch".tw.	209
73	Withings.tw.	58
74	Sensewear.tw.	525
75	PowerWatch.tw.	0
76	"Samsung Galaxy Watch".tw.	4
77	Airofit.tw.	3
78	Amazfit.tw.	5
79	VivaLINK.tw.	0
80	"Wellue DuoEK".tw.	0
81	KardiaMobile.tw.	31
82	"Philips Biosensor".tw.	1
83	Biovitals.tw.	4
84	exp Mood Disorder/	103245
85	mood disorder*.tw.	230732
86	exp Depression/	141189
87	depress*.tw.	518893
88	exp Stress, Psychological/	147280
89	stress*.tw.	972389
90	exp Psychological Distress/	5901
91	distress*.tw.	148661
92	1 or 2 or 3 or 4 or 5 or 6 or 7 or 8 or 9 or 10 or 11 or 12 or 13 or 14 or 15 or 16 or 17 or 18 or 19 or 20 or 21 or 22 or 23 or 24 or 25 or 26 or 27 or 28 or 29	233142
93	30 or 31 or 32 or 33 or 34 or 35 or 36 or 37 or 38 or 39 or 40 or 41 or 42 or 43 or 44 or 45 or 46 or 47 or 48 or 49 or 50 or 51 or 52 or 53 or 54 or 55 or 56 or 57 or 58 or 59 or 60 or 61 or 62 or 63 or 64 or 65 or 66 or 67 or 68 or 69 or 70 or 71 or 72 or 73 or 74 or 75 or 76 or 77 or 78 or 79 or 80 or 81 or 82 or 83	57765
94	84 or 85 or 86 or 87 or 88 or 89 or 90 or 91	1709010
95	92 and 93 and 94	243
96	limit 95 to (english language and yr="2015 -Current")	225

Database(s): **Embase** 1996 to 2022 Week 41.

#	Searches	Results
1	exp Artificial Intelligence/	60065
2	"Artificial Intelligence".tw.	22589
3	exp Machine Learning/	307253
4	"Machine Learning".tw.	65455
5	exp Deep Learning/	24222
6	"Deep Learning".tw.	30305
7	"supervised learning".tw.	4168
8	"unsupervised learning".tw.	1992
9	"reinforcement learning".tw.	4871
10	"Decision tree".tw.	14273
11	"K-Nearest Neighbor*".tw.	4679
12	"Support vector machine*".tw.	25140
13	"Recurrent neural network*".tw.	3588
14	"convolutional neural network*".tw.	18175
15	"Artificial neural network*".tw.	16577
16	"Deep Neural Networks".tw.	3396
17	"Naïve Bayes".tw.	17
18	"Naive Bayes".tw.	3021
19	"Fuzzy Logic".tw.	2679
20	"K-Means".tw.	7642
21	"Random Forest".tw.	16726
22	"Long Short-Term Memory Networks".tw.	308
23	autoencoder.tw.	1838
24	"boltzmann machine".tw.	464
25	"deep belief network".tw.	458
26	"Gradient Boost*".tw.	3835
27	AdaBoost.tw.	1324
28	"Multilayer Perceptron".tw.	2237
29	"Ensemble learning".tw.	1474
30	exp Wearable Electronic Devices/	6513
31	wearable*.tw.	21167
32	"smart watch*".tw.	261
33	smartwatch*.tw.	928
34	acceleromet*.tw.	24388
35	gyroscop*.tw.	2214
36	"inertial sensor".tw.	1232
37	"inertial measurement unit*".tw.	2475
38	"fitness band*".tw.	24
39	"flexible band*".tw.	268
40	headband*.tw.	518
41	"head band*".tw.	90
42	wristband*.tw.	1024
43	"smart insole*".tw.	180
44	"Smart armband".tw.	1
45	bracelet*.tw.	936
46	Emotiv.tw.	191
47	NeuroSky.tw.	149

48	Mindo.tw.	146
49	StarLab.tw.	6
50	EmSense.tw.	0
51	"B-Alert X24".tw.	0
52	Enobio.tw.	14
53	BrainBit.tw.	0
54	NeuroSky.tw.	18
55	Muse.tw.	1223
56	OpenBCI.tw.	21
57	Neuroelectrics.tw.	42
58	"G.tec nautilus".tw.	0
59	BioSemi.tw.	95
60	"mBrainTrain".tw.	1
61	Cognionics.tw.	8
62	"CGX QUICK".tw.	0
63	Fitbit.tw.	1473
64	Garmin.tw.	300
65	"Misfit shine".tw.	17
66	"Polar loop".tw.	28
67	Jawbone.tw.	825
68	Geneactiv.tw.	216
69	Empatica.tw.	83
70	Amiigo.tw.	3
71	Actigraph.tw.	4977
72	"Apple Watch".tw.	319
73	Withings.tw.	91
74	Sensewear.tw.	1075
75	PowerWatch.tw.	0
76	"Samsung Galaxy Watch".tw.	4
77	Airofit.tw.	1
78	Amazfit.tw.	6
79	VivaLINK.tw.	1
80	"Wellue DuoEK".tw.	0
81	KardiaMobile.tw.	52
82	"Philips Biosensor".tw.	2
83	Biovitals.tw.	3
84	exp Mood Disorder/	225092
85	mood disorder*.tw.	299011
86	exp Depression/	491389
87	depress*.tw.	575158
88	exp Stress, Psychological/	161707
89	stress*.tw.	1094813
90	exp Psychological Distress/	53522
91	distress*.tw.	184648
92	1 or 2 or 3 or 4 or 5 or 6 or 7 or 8 or 9 or 10 or 11 or 12 or 13 or 14 or 15 or 16 or 17 or 18 or 19 or 20 or 21 or 22 or 23 or 24 or 25 or 26 or 27 or 28 or 29	372011
93	30 or 31 or 32 or 33 or 34 or 35 or 36 or 37 or 38 or 39 or 40 or 41 or 42 or 43 or 44 or 45 or 46 or 47 or 48 or 49 or 50 or 51 or 52 or 53 or 54 or 55 or 56 or 57 or 58 or 59 or 60 or 61 or 62 or 63 or 64 or 65 or 66 or 67 or 68 or 69 or 70 or 71 or 72 or 73 or 74 or 75 or 76 or 77 or 78 or 79 or 80 or 81 or 82 or 83	59418
94	84 or 85 or 86 or 87 or 88 or 89 or 90 or 91	2046432
95	92 and 93 and 94	353

96	limit 95 to (english language and yr="2015 -Current")	315
97	limit 96 to exclude medline journals	68

Database(s): **APA PsycInfo** 2002 to October Week 1 2022.

#	Searches	Results
1	exp Artificial Intelligence/	23781
2	"Artificial Intelligence".tw.	5106
3	exp Machine Learning/	12327
4	"Machine Learning".tw.	9660
5	exp Deep Learning/	0
6	"Deep Learning".tw.	2682
7	"supervised learning".tw.	1105
8	"unsupervised learning".tw.	728
9	"reinforcement learning".tw.	3117
10	"Decision tree".tw.	1395
11	"K-Nearest Neighbor*".tw.	577
12	"Support vector machine*".tw.	3176
13	"Recurrent neural network*".tw.	1081
14	"convolutional neural network*".tw.	1237
15	"Artificial neural network*".tw.	2025
16	"Deep Neural Networks".tw.	647
17	"Naïve Bayes".tw.	7
18	"Naive Bayes".tw.	605
19	"Fuzzy Logic".tw.	846
20	"K-Means".tw.	1536
21	"Random Forest".tw.	1111
22	"Long Short-Term Memory Networks".tw.	27
23	autoencoder.tw.	328
24	"boltzmann machine".tw.	134
25	"deep belief network".tw.	65
26	"Gradient Boost*".tw.	215
27	AdaBoost.tw.	341
28	"Multilayer Perceptron".tw.	118
29	"Ensemble learning".tw.	152
30	exp Wearable Electronic Devices/	0
31	wearable*.tw.	2008
32	"smart watch*".tw.	45
33	smartwatch*.tw.	145
34	acceleromet*.tw.	4211
35	gyroscop*.tw.	108
36	"inertial sensor".tw.	53
37	"inertial measurement unit*".tw.	132
38	"fitness band*".tw.	2
39	"flexible band*".tw.	2
40	headband*.tw.	53
41	"head band*".tw.	3
42	wristband*.tw.	121
43	"smart insole*".tw.	1
44	"Smart armband".tw.	0
45	bracelet*.tw.	96

46	Emotiv.tw.	28
47	NeuroSky.tw.	13
48	Mindo.tw.	0
49	StarLab.tw.	1
50	EmSense.tw.	0
51	"B-Alert X24".tw.	0
52	Enobio.tw.	2
53	BrainBit.tw.	0
54	NeuroSky.tw.	13
55	Muse.tw.	301
56	OpenBCI.tw.	2
57	Neuroelectrics.tw.	1
58	"G.tec nautilus".tw.	0
59	BioSemi.tw.	15
60	"mBrainTrain".tw.	0
61	Cognionics.tw.	1
62	"CGX QUICK".tw.	0
63	Fitbit.tw.	245
64	Garmin.tw.	28
65	"Misfit shine".tw.	4
66	"Polar loop".tw.	1
67	Jawbone.tw.	23
68	Geneactiv.tw.	21
69	Empatica.tw.	56
70	Amiigo.tw.	0
71	Actigraph.tw.	1076
72	"Apple Watch".tw.	31
73	Withings.tw.	7
74	Sensewear.tw.	98
75	PowerWatch.tw.	0
76	"Samsung Galaxy Watch".tw.	0
77	Airofit.tw.	0
78	Amazfit.tw.	1
79	VivaLINK.tw.	0
80	"Wellue DuoEK".tw.	0
81	KardiaMobile.tw.	1
82	"Philips Biosensor".tw.	0
83	Biovitals.tw.	0
84	exp Mood Disorder/	55645
85	mood disorder*.tw.	155082
86	exp Depression/	9914
87	depress*.tw.	241746
88	exp Stress, Psychological/	0
89	stress*.tw.	209395
90	exp Psychological Distress/	0
91	distress*.tw.	66195
92	1 or 2 or 3 or 4 or 5 or 6 or 7 or 8 or 9 or 10 or 11 or 12 or 13 or 14 or 15 or 16 or 17 or 18 or 19 or 20 or 21 or 22 or 23 or 24 or 25 or 26 or 27 or 28 or 29	36989
93	30 or 31 or 32 or 33 or 34 or 35 or 36 or 37 or 38 or 39 or 40 or 41 or 42 or 43 or 44 or 45 or 46 or 47 or 48 or 49 or 50 or 51 or 52 or 53 or 54 or 55 or 56 or 57 or 58 or 59 or 60 or 61 or 62 or 63 or 64 or 65 or 66 or 67 or 68 or 69 or 70 or 71 or 72 or 73 or 74 or 75 or 76 or 77 or 78 or 79 or 80 or 81 or 82 or 83	7423

94	84 or 85 or 86 or 87 or 88 or 89 or 90 or 91	506879
95	92 and 93 and 94	53
96	limit 95 to (english language and yr="2015 -Current")	42

CINHAL: Monday, October 3, 2022 4:10:26 PM

#	Query	Results
S91	Narrow by Language: - english	29
S90	Limiters - Date Published: 20150101-20221231	29
S89	S86 AND S87 AND S88	29
S88	(S78 OR S79 OR S80 OR S81 OR S82 OR S83 OR S84 OR S85)	487,103
S87	(S30 OR S31 OR S32 OR S33 OR S34 OR S35 OR S36 OR S37 OR S38 OR S39 OR S40 OR S41 OR S42 OR S43 OR S44 OR S45 OR S46 OR S47 OR S48 OR S49 OR S50 OR S51 OR S52 OR S53 OR S54 OR S55 OR S56 OR S57 OR S58 OR S59 OR S60 OR S61 OR S62 OR S63 OR S64 OR S65 OR S66 OR S67 OR S68 OR S69 OR S70 OR S71 OR S72 OR S73 OR S74 OR S75 OR S76 OR S77)	13,841
S86	(S1 OR S2 OR S3 OR S4 OR S5 OR S6 OR S7 OR S8 OR S9 OR S10 OR S11 OR S12 OR S13 OR S14 OR S15 OR S16 OR S17 OR S18 OR S19 OR S20 OR S21 OR S22 OR S23 OR S24 OR S25 OR S26 OR S27 OR S28 OR S29)	25,112
S85	AB distress	53,231
S84	MW distress	18,307
S83	AB stress	142,755
S82	MW stress	154,999
S81	AB depress*	148,574
S80	MW depression	138,522
S79	AB Mood Disorder*	87,122
S78	MW Mood Disorder	69,864
S77	AB Biovitals	2
S76	AB "Philips Biosensor"	0
S75	AB KardiaMobile	13
S74	AB "Wellue DuoEK"	0
S73	AB VivaLINK	0
S72	AB Amazfit	1
S71	AB Airofit	0
S70	AB "Samsung Galaxy Watch"	2

S69	AB PowerWatch	1
S68	AB Sensewear	237
S67	AB Withings	43
S66	AB "Apple Watch"	115
S65	AB Actigraph	1,779
S64	AB Amiigo	0
S63	AB Empatica	74
S62	AB Geneactiv	87
S61	AB Jawbone	230
S60	AB "Polar loop"	5
S59	AB "Misfit shine"	9
S58	AB Garmin	92
S57	AB Garmin	0
S56	AB Fitbit	459
S55	AB "CGX QUICK"	0
S54	AB Cognionics	0
S53	AB "mBrainTrain"	0
S52	AB BioSemi	4
S51	AB "G.tec nautilus"	0
S50	AB Neuroelectrics	46
S49	AB Mindo	0
S48	AB NeuroSky	3
S47	AB Emotiv	12
S46	AB bracelet*	254
S45	AB "armband*"	280
S44	AB "Smart armband*"	0
S43	AB "smart headband*"	0
S42	AB "smart insole*"	1
S41	AB wristband*	248
S40	AB "head band*"	21

S39	AB headband*	102
S38	AB "fitness band*"	12
S37	AB "inertial measurement unit*"	418
S36	AB "inertial sensor"	196
S35	AB gyroscop	0
S34	AB acceleromet*	7,796
S33	AB smartwatch*	189
S32	AB "smart watch*"	59
S31	AB wearable*	3,069
S30	MW wearable devices	91
S29	AB "Ensemble learning"	103
S28	AB "Multilayer Perceptron"	195
S27	AB AdaBoost	131
S26	AB "Gradient Boost*"	564
S25	AB "deep belief network"	24
S24	AB "boltzmann machine"	10
S23	AB autoencoder	93
S22	AB "Long Short-Term Memory Networks"	15
S21	AB "Random Forest"	2,006
S20	AB "K-Means"	972
S19	AB "Fuzzy Logic"	207
S18	AB "Naive Bayes"	175
S17	AB "Naïve Bayes"	256
S16	AB "Deep Neural Networks"	147
S15	AB "Artificial neural network*"	1,195
S14	AB "convolutional neural network*"	1,359
S13	AB "Recurrent neural network*"	182
S12	AB "Support vector machine*"	2,429
S11	AB "K-Nearest Neighbor*"	412
S10	AB "Decision tree"	2,352

S9	AB "reinforcement learning"	272
S8	AB "unsupervised learning"	107
S7	AB "supervised learning"	250
S6	AB "deep Learning"	2,688
S5	MW "deep Learning"	999
S4	AB "Machine Learning"	7,913
S3	MW "Machine Learning"	3,155
S2	AB "Artificial Intelligence"	4,314
S1	MW Artificial Intelligence	7,059

Database	Query	Results
**Scopus**	(TITLE-ABS-KEY ("Artificial Intelligence" OR "Machine Learning" OR "Deep Learning" OR "supervised learning" OR "unsupervised learning" OR "reinforcement learning" OR "Decision tree" OR "K-Nearest Neighbor*" OR "Support vector machine*" OR "Recurrent neural network*" OR "convolutional neural network*" OR "Artificial neural network*" OR "Deep Neural Networks" OR "Naïve Bayes" OR "Naive Bayes" OR "Fuzzy Logic" OR "K-Means" OR "Random Forest" OR "Long Short-Term Memory Networks" OR autoencoder OR "boltzmann machine" OR "deep belief network" OR "Gradient Boost*" OR adaboost OR "Multilayer Perceptron" OR "Ensemble learning")) AND (TITLE-ABS-KEY (wearable* OR "smart watch*" OR smartwatch* OR acceleromet* OR gyroscop* OR "inertial sensor" OR "inertial measurement unit*" OR "fitness band*" OR "flexible band*" OR headband* OR "head band*" OR wristband* OR "smart insole*" OR "Smart armband" OR bracelet* OR emotiv OR neurosky OR mindo OR starlab OR emsense OR "B-Alert X24" OR enobio OR brainbit OR muse OR openbci OR neuroelectrics OR "G.tec nautilus" OR biosemi OR "mBrainTrain" OR cognionics OR "CGX QUICK" OR fitbit OR garmin OR "Misfit shine" OR "Polar loop" OR jawbone OR geneactiv OR empatica OR amiigo OR actigraph OR "Apple Watch" OR withings OR sensewear OR powerwatch OR "Samsung Galaxy Watch" OR airofit OR amazfit OR vivalink OR "Wellue DuoEK" OR kardiamobile OR "Philips Biosensor" OR biovitals)) AND (TITLE-ABS-KEY (mood disorder* OR depress* OR stress* OR distress)) AND (LIMIT-TO (LANGUAGE , "English")) AND (LIMIT-TO (PUBYEAR , 2022) OR LIMIT-TO (PUBYEAR , 2021) OR LIMIT-TO (PUBYEAR , 2020) OR LIMIT-TO (PUBYEAR , 2019) OR LIMIT-TO (PUBYEAR , 2018) OR LIMIT-TO (PUBYEAR , 2017) OR LIMIT-TO (PUBYEAR , 2016) OR LIMIT-TO (PUBYEAR , 2015)) AND (LIMIT-TO (DOCTYPE , "ar") OR LIMIT-TO (DOCTYPE , "cp") OR LIMIT-TO (DOCTYPE , "ch"))	775

**IEEE Xplore**	("Abstract":"Artificial Intelligence" OR "Abstract":"Machine Learning" OR "Abstract":"Deep Learning" OR "Abstract":"supervised learning" OR "Abstract":"unsupervised learning" OR "Abstract":"reinforcement learning" OR "Abstract":"Decision tree" OR "Abstract":"K-Nearest Neighbor" OR "Abstract":"Support vector machine" OR "Abstract":"Recurrent neural network" OR "Abstract":"convolutional neural network" OR "Abstract":"Artificial neural network" OR "Abstract":"Deep Neural Network" OR "Abstract":"Naïve Bayes" OR "Abstract":"Naive Bayes" OR "Abstract":"Fuzzy Logic" OR "Abstract":"K-Means" OR "Abstract":"Random Forest" OR "Abstract":"Long Short-Term Memory Networks" OR "Abstract":autoencoder OR "Abstract":"boltzmann machine" OR "Abstract":"deep belief network" OR "Abstract":"Gradient Boost" OR "Abstract":AdaBoost OR "Abstract":"Multilayer Perceptron" OR "Abstract":"Ensemble learning") AND ("Abstract":wearable OR "Abstract":wearable OR "Abstract":"smart watch" OR "Abstract":smartwatch OR "Abstract":"smart watches" OR "Abstract":smartwatches OR "Abstract":acceleromet OR "Abstract":gyroscop OR "Abstract":"inertial sensor" OR "Abstract":"inertial measurement unit" OR "Abstract":"fitness band" OR "Abstract":"flexible band" OR "Abstract":headband OR "Abstract":"head band" OR "Abstract":wristband OR "Abstract":"smart insole" OR "Abstract":"Smart armband" OR "Abstract":bracelet* OR "Abstract":Emotiv OR "Abstract":NeuroSky OR "Abstract":Mindo StarLab OR "Abstract":EmSense OR "Abstract":"B-Alert X24" OR "Abstract":Enobio OR "Abstract":BrainBit OR "Abstract":Muse OR "Abstract":OpenBCI OR "Abstract":Neuroelectrics OR "Abstract":"G.tec nautilus" OR "Abstract":BioSemi OR "Abstract":"mBrainTrain" OR "Abstract":Cognionics OR "Abstract":"CGX QUICK" OR "Abstract":Fitbit OR "Abstract":Garmin OR "Abstract":"Misfit shine" OR "Abstract":"Polar loop" OR "Abstract":Jawbone OR "Abstract":Geneactiv OR "Abstract":Empatica OR "Abstract":Amiigo OR "Abstract":Actigraph OR "Abstract":"Apple Watch" OR "Abstract":Withings OR "Abstract":Sensewear OR "Abstract":PowerWatch OR "Abstract":"Samsung Galaxy Watch" OR "Abstract":Airofit OR "Abstract":Amazfit OR "Abstract":VivaLINK OR "Abstract":"Wellue DuoEK" OR "Abstract":KardiaMobile OR "Abstract":"Philips Biosensor" OR "Abstract":Biovitals) AND ("Abstract": mood disorder* OR "Abstract":depress* OR "Abstract":stress* or distress)	35
**ACM Digital library**	[[Abstract: "artificial intelligence"] OR [Abstract: "machine learning"] OR [Abstract: "deep learning"] OR [Abstract: "supervised learning"] OR [Abstract: "unsupervised learning"] OR [Abstract: "reinforcement learning"] OR [Abstract: "decision tree"] OR [Abstract: "k-nearest neighbor*"] OR [Abstract: "support vector machine*"] OR [Abstract: "recurrent neural network*"] OR [Abstract: "convolutional neural network*"] OR [Abstract: "artificial neural network*"] OR [Abstract: "deep neural networks"] OR [Abstract: "naïve bayes"] OR [Abstract:	40

	"naive bayes"] OR [Abstract: "fuzzy logic"] OR [Abstract: "k-means"] OR [Abstract: "random forest"] OR [Abstract: "long short-term memory networks"] OR [Abstract: autoencoder] OR [Abstract: "boltzmann machine"] OR [Abstract: "deep belief network"] OR [Abstract: "gradient boost*"] OR [Abstract: adaboost] OR [Abstract: "multilayer perceptron"] OR [Abstract: "ensemble learning"]] AND [[Abstract: wearable*] OR [Abstract: "smart watch*"] OR [Abstract: smartwatch*] OR [Abstract: acceleromet*] OR [Abstract: gyroscop*] OR [Abstract: "inertial sensor"] OR [Abstract: "inertial measurement unit*"] OR [Abstract: "fitness band*"] OR [Abstract: "flexible band*"] OR [Abstract: headband*] OR [Abstract: "head band*"] OR [Abstract: wristband*] OR [Abstract: "smart insole*"] OR [Abstract: "smart armband"] OR [Abstract: bracelet*] OR [Abstract: emotiv] OR [Abstract: neurosky] OR [Abstract: mindo starlab] OR [Abstract: emsense] OR [Abstract: "b-alert x24"] OR [Abstract: enobio] OR [Abstract: brainbit] OR [Abstract: muse] OR [Abstract: openbci] OR [Abstract: neuroelectrics] OR [Abstract: "g.tec nautilus"] OR [Abstract: biosemi] OR [Abstract: "mbraintrain"] OR [Abstract: cognionics] OR [Abstract: "cgx quick"] OR [Abstract: fitbit] OR [Abstract: garmin] OR [Abstract: "misfit shine"] OR [Abstract: "polar loop"] OR [Abstract: jawbone] OR [Abstract: geneactiv] OR [Abstract: empatica] OR [Abstract: amiigo] OR [Abstract: actigraph] OR [Abstract: "apple watch"] OR [Abstract: withings] OR [Abstract: sensewear] OR [Abstract: powerwatch] OR [Abstract: "samsung galaxy watch"] OR [Abstract: airofit] OR [Abstract: amazfit] OR [Abstract: vivalink] OR [Abstract: "wellue duoek"] OR [Abstract: kardiamobile] OR [Abstract: "philips biosensor"] OR [Abstract: biovitals]] AND [[Abstract: mood disorder*] OR [Abstract: depress*] OR [Abstract: stress* or distress]] AND [Publication Date: (01/01/2015 TO 12/31/2022)]	
**Google Scholar**	("Artificial Intelligence" OR "Machine Learning" OR "Deep Learning") AND (wearable* OR smartwatch* OR Emotiv OR Mindo OR Muse OR Fitbit OR Garmin OR Geneactiv OR Empatica "Apple Watch" OR "Polar loop") AND (mood disorder* OR depress* OR stress* OR distress)	100

Supplementary Table 12: Data extraction form

Extracted data	Definition
**Study Characteristics**	
Author	The first author of the study.
Year of publication	The year in which the study was published.
Country of publication	The country where the study was published.
Type of publication	The venue where the study was published: peer-reviewed journal articles, book chapters, dissertations, or conference proceedings
Number of participants	What is the number of participants from which the data was collected?
Mean age (range)	What is the mean/range age of the participants?
Female percentage	What is the female percentage of the participants?
Participants Health Conditions	What is the health condition of the participants?
**Wearable AI characteristics**	
Name of the wearable device	What is the name of the wearable device (e.g., Fitbit, Empatica, ApplyWatch, ActiWatch, etc..)?
Placement of the wearable device	Where the wearable device is worn during the experiment in paper or normally (wrist, chest, head, ears, forehead, eyes, fingers, foot, etc..)?
Aim of AI algorithm	What was the algorithm used for (diagnosis, screening, monitoring, treatment, prevention, etc.))?
Problem solving approaches	What is the problem-solving approach that the algorithm follows (Classification, regression)?
AI algorithm used	What are the main AI algorithms/models (e.g., RF, SVM, ANN, CNN, RNN, DNN, k-NN, MLP, DBN, DBM, DPN BN, CRT, DT, LASSO, LR, MFA, MLR, MDL, NB, NN, NSC, RBFN) used in the paper?
Data sources	What is the source of data that was used for developing the algorithms (open source or closed source)?
Data input	What is the data that was used for developing the algorithm?
Ground truth assessment	How the actual status (e.g., diagnosis) of the user was confirmed (questionnaire (PHQ-9), interview, test, etc..)?
Type of validation	What is the approach that was used to validate the developed algorithm (e.g., Training-test split, K-fold cross-validation, Nested Cross-Validation, Leave One Out cross-validation, Apparent validation, external validation)?
Performance measures used	What are the measures used to assess the performance of the algorithm (accuracy, sensitivity (recall), specificity, precision, AUC, etc...)?
Results	The highest and lowest results for each performance measure for each algorithm. Calculate the measures if the confusion matrix is reported.

Supplementary Table 13: The modified version of the Quality of Diagnostic Accuracy Studies 2 (QUADAS-2)

Participants	Signaling questions	Explanation
	1.1 Was a consecutive or random sample of patients enrolled?	-Yes: if a consecutive or random sample of eligible patients was enrolled.<br><br>- No: if patients were selected by convenience;<br><br>- Unclear: if the study did not report the manner in which participants were enrolled.
	1.2 Did the study avoid inappropriate exclusions?	- Yes: If inclusion and exclusion of participants were appropriate, so participants correspond to unselected participants of interest.<br><br>- No: If participants are included who would already have been identified as having the outcome and so are no longer participants at suspicion of disease (diagnostic studies),<br>or if specific subgroups are excluded that may have altered the performance of the prediction model for the intended target population.<br><br>- Unclear: When there is no information on whether inappropriate inclusions or exclusions took place.
	1.3 Was the sample size sufficient?	- Yes: For model validation studies, if the number of participants is ≥100.<br><br>- No: For model validation studies, if the number of participants with the outcome is <100.<br><br>- Unclear: For model development studies, no information on the number of candidate predictor parameters or number of participants<br>with the outcome, such that the EPV cannot be calculated.<br>For model validation studies, no information on the number of participants with the outcome.
	1.4 Was there a balance in the number of patients between the subgroups (depressed vs. nondepressed)?	- Yes: if the percentage of participants in any group is 66.7 or less of the sample (≤2/3).<br><br>- No: if the percentage of participants in any group is more than 66.7 of the sample (>2/3).<br><br>- Unclear: If no information was provided regarding the number of participants in the groups.
	**Risk-of-bias assessment:** Could the selection of	- Low risk of bias: If the answer to all signaling questions is 'Yes' then the risk of bias can be

	participants have introduced bias?	considered low. If one or more of the answers is 'No', the judgment could still be low risk of bias, but specific reasons why the risk of bias can be considered low should be provided.<br><br>- High risk of bias: If the answer to any of the signaling questions is "No" there is a potential for bias, except if defined at low risk of bias above.<br><br>- Unclear risk of bias: If relevant information is missing for all or some of the signaling questions, and none of the answers to signaling questions is judged to put this domain at high risk of bias.
	**Concerns regarding applicability:** Are there concerns that the included participants and setting do not match the review question?	- Low concern for applicability: If the spectrum of participants (in- and exclusion criteria, setting, prior testing) matches the pre-stated requirements in the review question<br><br>- High concern for applicability: If the spectrum of participants does not fully match the pre-stated requirements in the review question<br><br>- Unclear concern for applicability: If there is insufficient information available to make a judgment about the applicability
**Index test (AI algorithms)**	2.1 Were the AI models described in detail?	-Yes: if the model details were provided such as outputs, epoch, all intermediate layers and connections, pooling, normalization, regularization, and activation in the layers, etc.<br>or if a previously published model is<br>employed, the paper must cite a reference that meets the preceding standards and fully describe every modification made to the model.<br><br>-No: if only the model's name was reported in the paper, or the study reported some information but other important information still missing.
	2.2 Were all features (predictors) used in the model clearly identified?	-Yes: If all features (e.g., heart rate, inter-beat interval, heart rate variation, number of sleep hours, etc.) used in each model were reported.<br><br>-No: If any features used in any model were not reported.<br>Or all features used in the paper were identified but it was not clear which features were used in each model.

	2.3 Were features assessed in the same way for all participants?	Please notice some studies used different wearable devices to collect the data.<br><br>-Yes: If the assessment of features assessment were similar for all participants.<br><br>-No: If different definitions were used for the same predictor or if predictors requiring subjective interpretation were assessed by differently experienced assessors.<br><br>Unclear: If there is no information on how predictors were defined or assessed.
	2.4 Were features collected without knowledge of outcome data (depression status)?	- Yes: If outcome information was stated as not used during feature assessment or was clearly not (yet) available to those assessing features.<br><br>- No: If it is clear that outcome information was used when assessing predictors.<br><br>- Unclear: No information on whether features were assessed without knowledge of outcome information.
	**Risk-of-bias assessment:** Could the conduct or interpretation of the index test have introduced bias?	- Low risk of bias: If the answer to all signaling questions is 'Yes' then the risk of bias can be considered low. If one or more of the answers is 'No', the judgment could still be low risk of bias, but specific reasons why the risk of bias can be considered low should be provided e.g., the use of objective predictors not requiring subjective interpretation.<br><br>- High risk of bias: If the answer to any of the signaling questions is "No" there is a potential for bias, except if defined at low risk of bias above.<br><br>- Unclear risk of bias: If relevant information is missing for all or some of the signaling questions, and none of the answers to signaling questions is judged to put this domain at high risk of bias.
	**Concerns regarding applicability:** Are there concerns that the definition, assessment, or timing of the index test in the model does not match the review question?	- Low concern for applicability: Definition, assessment, and timing of predictors match the review question.<br><br>- High concern for applicability: Definition, assessment, or timing of predictors were different from the review question.

		- Unclear concern for applicability: If relevant information about the predictors is not reported.
**Reference Standard (Ground truth)**	3.1 Was the reference standard likely to correctly classify the outcome (e.g., depressed vs. non-depressed)?	Is the used tool appropriate?<br><br>Any tool recommended by APA: BDI, PHQ-9, DSM-IV, DSM-5, HAM-D, GDS, MADRS, CDI, CDRS, BHS, CES-D, EQ-5D, BASC, CBCL, QIDS-SR, RFS, SF-36. Or any tool has reliability and validity of >=0.70.<br><br>Were the assessors/annotators qualified?<br><br>- Yes: If the study used well-recommended tools such as BDI, PHQ-9, DSM-IV, DSM-5, HAM-D, GDS, MADRS, CDI, CDRS, BHS, CES-D, EQ-5D, BASC, CBCL, QIDS-SR, RFS, SF-36.<br>Or any tool has reliability and validity of >=0.70.<br>Or an interview was conducted by a qualified assessor such as a psychologist or psychiatrist<br><br>-No: if the outcome was assessed using only one question (e.g., how depressed do you feel today?). OR Unknown questionnaire with reliability and validity of <0.70 or unknown reliability and validity. OR an interview was conducted by unqualified assessors such as students.<br><br>- Unclear: If no information was provided about the reference standard
	3.2 Was the outcome defined and determined in a similar way for all participants?	- Yes: If outcomes were defined and determined in a similar way for all participants.<br>- No: If outcomes were clearly defined and determined in a different way for some participants.<br>- Unclear: No information on whether outcomes were defined or determined in a similar way for all participants.
	3.3 Was the outcome determined without knowledge of predictor information?	- Yes: If predictor information was not known when determining the outcome status, or outcome status determination is clearly reported as determined without knowledge of predictor information.<br>- No: If it is clear that predictor information was used when determining the outcome status.<br>- Unclear: No information on whether the outcome was determined without knowledge of predictor information.
	3.4 Was there an appropriate interval	Check the period in which the reference standard assesses symptoms of depression. For example, PHQ-9, HDRS-D, MADRS, DASS, GDS, and BDI assess

	between the index test and the reference standard?	symptoms of depression experienced over the past week. When such tools are used, then the interval between the index test and reference standard should not be more than 7 days.<br><br>BDI-II: over the past 2 weeks<br><br>- Yes: If the time interval between predictor assessment and outcome determination was appropriate to enable the correct type and representative number of relevant outcomes to be recorded, or if no information on the time interval is required to allow a representative number of the relevant outcome occur or if predictor assessment and outcome determination were from information taken within an appropriate time interval.<br><br>- No: If the time interval between predictor assessment and outcome determination is too short or too long to enable the correct type and representative number of relevant outcomes to be recorded.<br><br>- Unclear: If no information was provided on the time interval between predictor assessment and outcome determination.
	**Risk-of-bias assessment:** Could the reference standard, its conduct, or its interpretation have introduced bias?	- Low risk of bias: If the answer to all signaling questions is 'Yes' then the risk of bias can be considered low. If one or more of the answers is 'No', the judgment could still be low risk of bias, but specific reasons why the risk of bias can be considered low should be provided e.g., when the outcome was<br>determined with knowledge of predictor information but the outcome assessment did not require much interpretation by the assessor (e.g., death regardless of cause).<br><br>- High risk of bias: If the answer to any of the signaling questions is "No" there is a potential for bias, except if defined at low risk of bias above.<br><br>- Unclear risk of bias: If relevant information is missing for all or some of the signaling questions, and none of the answers to signaling questions is judged to put this domain at high risk of bias.

	**Concerns regarding applicability:** Are there concerns that the outcome definition, timing, or determination do not match the review question?	- Low concern for applicability: Outcome definition, timing, and method of determination defines the outcome as intended by the review question.<br><br>-High concern for applicability: Choice of outcome definition, timing, and method of outcome determination defines another outcome as intended by the review question.<br><br>- Unclear concern for applicability: If relevant information about the outcome, timing, and method of determination is not reported.
**Analysis**	4.1 Were all participants included in the analysis?	- Yes: If all participants enrolled in the study are included in the data analysis.<br><br>- No: If some or a subgroup of participants are inappropriately excluded from the analysis.<br><br>- Unclear: No information on whether all enrolled participants are included in the analysis.
	4.2 Was data preprocessing carried out appropriately?	- Yes: If there are no missing values of predictors or outcomes and the study explicitly reports that participants are not excluded on the basis of missing data, or if missing values are handled using multiple imputation.<br><br>- No: If participants with missing data are omitted from the analysis, or if the method of handling missing data is clearly flawed, e.g., missing indicator method or inappropriate use of last value carried forward, or if the study had no explicit mention of methods to handle missing data.<br><br>- Unclear: If there is insufficient information to determine if the method of handling missing data is appropriate.
	4.3 Was the breakdown of the training, validation, and test sets appropriate?	- Yes: if the study used an appropriate validation approach.<br><br>- No: if the study used an inappropriate validation approach.<br><br>- Unclear: If no information was provided about the validation methods.
	4.4 Was the performance of the model evaluated appropriately?	- Yes: If the confusion matrix was presented,<br>Or more than one measure was used and the selected measures were appropriate.

		- No: If the confusion matrix was not presented, and only one measure was reported,<br>Or the selected measures were not appropriate.<br><br>- Unclear: If no information was provided on the performance measures
	**Risk-of-bias assessment:** Could the analysis, its conduct, or its interpretation have introduced bias?	- Low risk of bias: If the answer to all signaling questions is 'Yes' then the risk of bias can be considered low. If one or more of the answers is 'No', the judgment could still be low risk of bias, but specific reasons why the risk of bias can be considered low should be provided.<br><br>- High risk of bias: If the answer to any of the signaling questions is "No" there is a potential for bias, except if defined at low risk of bias above.<br><br>- Unclear risk of bias: If relevant information is missing for all or some of the signaling questions, and none of the answers to signaling questions is judged to put the analysis at high risk of bias.
